# Real-time driver drowsiness detection using transformer architectures: a novel deep learning approach

**DOI:** 10.1038/s41598-025-02111-x

**Published:** 2025-05-20

**Authors:** Osama F. Hassan, Ahmed F. Ibrahim, Ahmed Gomaa, M. A. Makhlouf, B. Hafiz

**Affiliations:** 1 Information Systems Department, Faculty of Computers and Informatics, Suez Canal University, Ismailia, 41522 Egypt; 2 Artificial Intelligence Department, Faculty of Computer Science and Engineering, King Salman International University (KSIU), South Sinai, 46511 Egypt; 3 Computer Science and Engineering Department, Egypt Japan University of Science and Technology (E-JUST), Alexandria, 21934 Egypt; 4 Computer Science and Engineering Department, National Research Institute of Astronomy and Geophysics (NRIAG), Helwan, 11731 Egypt

**Keywords:** Computational science, Computer science

## Abstract

Driver drowsiness is a leading cause of road accidents, resulting in significant societal, economic, and emotional losses. This paper introduces a novel and robust deep learning-based framework for real-time driver drowsiness detection, leveraging state-of-the-art transformer architectures and transfer learning models to achieve unprecedented accuracy and reliability. The proposed methodology addresses key challenges in drowsiness detection by integrating advanced data preprocessing techniques, including image normalization, augmentation, and region-of-interest selection using Haar Cascade classifiers. We employ the MRL Eye Dataset to classify eye states into “Open-Eyes” and “Close-Eyes,” evaluating a range of models, including Vision Transformer (ViT), Swin Transformer, and fine-tuned transfer learning models such as VGG19, DenseNet169, ResNet50V2, InceptionResNetV2, InceptionV3, and MobileNet. The ViT and Swin Transformer models achieved groundbreaking accuracy rates of 99.15% and 99.03%, respectively, outperforming all other models in precision, recall, and F1-score. To ensure the generalization and robustness of the proposed models, we also evaluate their performance on the NTHU-DDD and CEW datasets, which provide diverse real-world scenarios and challenging conditions. This represents a significant advancement over existing methods, demonstrating the effectiveness of transformer-based architectures in capturing complex spatial dependencies and extracting relevant features for drowsiness detection. The proposed system also incorporates a real-time drowsiness scoring mechanism, which triggers alarms when prolonged eye closure is detected, ensuring timely intervention to prevent accidents. A key novelty of this work lies in the integration of Class Activation Mapping (CAM) for enhanced model interpretability, allowing the system to focus on critical eye regions and improve decision-making transparency. The system was rigorously tested under varying lighting conditions and scenarios involving glasses, showcasing its robustness and adaptability for real-world deployment. By combining cutting-edge deep learning techniques with real-time processing capabilities, this research offers a contactless, reliable, and efficient solution for driver drowsiness detection, significantly contributing to improved road safety and accident prevention. The proposed framework sets a new benchmark in drowsiness detection, highlighting its potential for widespread adoption in advanced driver assistance systems.

## Introduction

In the modern era, based on our ways of living, constant tiredness is more common than it has ever been. Today, an individual sleeps 20 percent less than what was normal a hundred years ago^1^. This remains a primary cause for the decline of performance across various tasks in daily life. Subsequently, this leads to people being less focused when engaging in simple activities. This does not change when it comes to driving. A driver who is exhausted suffers a great deal when it comes to performance, and this decline is even more pronounced in the absence of proper sleep. A person who does not want to suffer the negative consequences of being excessively tired simply needs to get sufficient sleep. For a healthy human adult, the average number of hours of recommended sleep is around 7 hours, but currently, 1 in 3 adults suffer from deprivation of this amount of sleep^2^. This is caused by the nature of today’s lifestyle. Most of these consequences stem from being excessively drowsy, and hence road accidents have seen an upward spike recently^3^. The aftermath of these accidents incurs a diverse range of societal, economic, and emotional losses.

From 2000 to 2016, the case fatality rate and human losses due to road accidents in China increased by 19.0% and 63.7%, respectively^4^. A 2002 survey in Ontario, Canada, showed that more than 58% of the 750 respondents confessed to driving while fatigued, with 14.5% of them dozing off at the wheel^5^. The economic costs linked to drowsy driving was estimated at $12.4 billion in a single year^6^. Recognition of these facts is the reason why it is recommended to put in place strategies that will guarantee traffic safety. Certain businesses such as Mercedes-Benz, Tesla, and others provide a variety of driver assistance systems^7,8^. For instance, automatic brakes, cruise control, lane change warning, assisted steering, and many others. This has greatly assisted in preventing road accidents and saving innocent lives. Nevertheless, the potential of a driver feeling drowsy or fatigued without any standard means of assessment poses a threat to safety on the road. Fatigue, often characterized by diminished alertness and slower reaction times, plays a critical role in the onset of drowsy driving and has been linked to increased accident risk. The brain activity patterns offer valuable physiological indicators of fatigue, reinforcing the importance of accurate detection in preventing accidents^9^.

For the successful averting of road accidents, detection of drowsiness is a fundamental need. So we were looking to figure out how to come up with a program that could guess when the driver was going to feel drowsy and notify him beforehand to avert a serious incident on the road. In working toward this goal, we need to keep track of the driver’s face and search for clues that may suggest that he is dozing off. To solve this problem, we applied deep learning methods, which are explained in the following sections.

To effectively detect and prevent drowsy driving, different approaches have been devised targeting and measuring driver performance, behavior, and physiological state. These approaches can typically be classified into biological, image or video-based, vehicle-based, and hybrid measurements. Each type captures signs of fatigue physiologically and behaviorally, as well as through vehicle dynamics, using internal and external measures. Different associations of each measurement type is provided in Table 1 and their definitions, representative examples, advantages, and limitations summarized. This type of classification illustrates the broad multi-dimensional driver drowsiness detection systems and methods currently available, just as much as it explains the ease with which systems can be developed or integrated within existing frameworks designed for future research and application.

**Table 1:** Overview of driver drowsiness detection measurement types.

Measurement type	Definition	Examples	Advantages	Limitations
Biological measurements	Use physiological signals to assess driver’s internal state and fatigue levels	Brain Signal, Respiratory Signal, Heart Signal, Skin Signal, eye signal	High accuracy in detecting true drowsiness; real-time monitoring of body functions	Intrusive; requires wearable sensors; may cause discomfort or distraction
Image-/video-based measurements	Analyze driver’s facial features and head posture via camera input	Eye, Mouth, Head, Hybrid (Eye, Mouth, Head)	Non-intrusive; cost-effective; compatible with deep learning and computer vision methods	Sensitive to lighting, occlusion, and sunglasses; high computational cost
Vehicle-based measurements	Derive behavioral data from vehicle dynamics and driver interaction	Steering Wheel, Lane Deviation	Non-intrusive; utilizes existing vehicle systems; no need for driver contact	Indirect measurement; affected by road conditions and driving style
Hybrid measurements	Combine two or more types to increase detection accuracy	Vehicle & Image, Biological & Image, Vehicle & Biological, Vehicle, Image & biological	Robust and reliable; complements strengths of individual methods	Complex system integration; increased cost and processing requirements

Recent advances in driver drowsiness detection have shifted from heuristic-based methods (e.g., PERCLOS, eye aspect ratio) to deep learning models, particularly CNNs like VGG16 and ResNet, which achieve 92–97% accuracy on benchmark datasets^10,11^. However, these models struggle with long-range spatial dependencies in facial features (e.g., subtle eyelid movements during micro-sleeps) due to their localized receptive fields. Transformers, with their self-attention mechanisms, address this by capturing global context-a critical advantage for drowsiness detection where holistic facial cues (e.g., brow furrowing, slow blinks) are as informative as local eye states. Despite their success in NLP and image classification, transformer-based drowsiness detection remains underexplored, with only preliminary studies like^12^ reporting ViT adaptations for this task. Our work bridges this gap by rigorously evaluating ViT and Swin Transformer architectures against state-of-the-art CNNs, demonstrating that transformers outperform CNNs not only in accuracy (99.15% vs. 98.7% for VGG19) but also in robustness to lighting variations. Unlike prior CNN-based systems^13–18^, our framework integrates Class Activation Mapping (CAM) to visually justify predictions-a critical step for real-world deployment where false alarms risk user distrust. Transformers excel in drowsiness detection due to their ability to model relationships between distant facial regions (e.g., correlating yawning with prolonged eye closure) via self-attention. This contrasts with CNNs, which require deeper architectures to achieve similar context awareness, often at the cost of computational efficiency. Our Swin Transformer variant further optimizes this by hierarchically processing windows of attention, reducing latency for real-time use.

This paper aims to develop a robust eye states classification of Open-Eyes and Close-Eyes based on a deep learning framework using the MRL dataset and deploy the best-performing model for real feedback accurate eye states. The proposed methodology incorporates a set sequence of steps starting with data preparation which involves adjusting the image size, normalization, and augmentation. Several deep learning practices are used which include transformer-based models such as Vision Transformer (ViT) and Swin Transformer, and for transfer learning, models such as VGG19, Attention VGG19, DenseNet169, ResNet50V2, InceptionResNetV2, InceptionV3, and MobileNet. From a set of specific criteria such as accuracy, precision, recall and F1-score, the best-performing model is selected after training and evaluation. The determined model is then fitted into a system that allows feeding real-time processed video frames that apply Haar Cascade classifiers to locate the face and the eyes of the driver, and subsequently, observe the position of the driver’s eyes in order to detect whether he or she is drowsy. An alarm is issued to the driver if a significant period of time is spent with the eyes closed. By leveraging state-of-the-art deep learning models and real-time processing techniques, this study provides a reliable and effective approach for driver drowsiness detection, contributing to improved road safety and accident prevention.

The key contributions of this work are as follows:

- Extensive model assessment: We performed model assessments utilizing various types of deep learning models which include transformer techniques and transfer learning algorithms for identifying Open-Eyes and Close-Eyes images from the MRL dataset.
- Optimized drowsiness detection pipeline: The drowsiness detection system has been enhanced whereby it now involves a sequence of activities such as data preparation, evaluation of the performance of the model, and visualization using Class Activation Mapping (CAM) for interpretability.
- Real-time implementation: A real-world setting where the optimal proposed model trained is used, the system uses a face and eye tracker that has an active score keeper to monitor the environment and sound alarms when people are drowsy.
- Enhanced road safety: It’s a contactless technology that can be deployed in-car driver assisting tools so that fatigue or drowsy driving can be decreased and accidents reduced.

The rest of the paper is structured as follows: In “Literature review” section, present a critical appraisal of related works. In “Proposed methodology” section, we discuss the methodology proposed. “Experiments and results” section presents the experimental results. “Comparative study” section presents the comprehensive study. In “Discussion” section, the discussion is presented. “Conclusion and future directions” section: concludes this paper.

## Literature review

Some researchers have worked on real-time eye state recognition systems that can be used in embedded devices with limited resources. In this respect, the authors in^19^ discuss the application of eye tracking data as an unobtrusive measure of driver behavior for detecting drowsiness. Tracking of eye movements was carried out for 53 participants in a driving simulation task, while channels of multichannel electroencephalogram (EEG) were obtained as baselines. Vigilance states are phase classified using a random forest (RF), and a binary classifier based on a nonlinear support vector machine (SVM). The extraction of features was done for various lengths of eye movement tracking epochs, and the assessment of each classifier was done for each epoch length. The RF classifier provided high accuracy irrespective of the epoch lengths ranging from 88.37% to 91.18%. The SVM classifier remained inferior with an accuracy of 77.12% to 82.62% at all epoch lengths.

DriCare is a system introduced by authors in^20^ which utilizes video images to detect the fatigue of drivers without the necessity to connect any devices to them. It uses yawning, blinking and the duration of eye closure as cues. The authors present a new tactic targeting the problems that previous algorithms exhibited, face tracking for increasing the tracking precision. Moreover, he introduces a new approach for detecting facial regions corresponding to 68 major facial characteristics. These regions are then utilized to evaluate the state of the driver. DriCare is able to warn a driver with a system message if there are features detected in the mouth or eyes that could indicate fatigue. DriCare gets a value of 92% directly according to published experimental results.

The authors in^21^ detailed the creation of an inexpensive portable apparatus that would evaluate the drowsiness level of a driver. This device is composed of an IR illuminator and a camera that sends the image to a Raspberry Pi 3 Model B. This configuration allows for a system that is less sensitive to changes in environmental light and vision blockage. The processing model takes upper face and oral movements into account (PERCLOS, ECD, and AOT) and establishes three levels of drowsiness based on fuzzy problems (Low-Normal State, Medium-Drowsy State, and High-Severe Drowsiness State). Two different methods of face feature recognition were tested: a facial cascade classifier with haar like filter features and a linear support vector machine SVM with HOG features.

A new suggestion regarding advanced facial thermal imaging technology able to analyze drivers’ respiration without any physical interaction is discussed in this paper^22^. During a car simulator experiment with thirty participants, respiration signals were gathered when participants were encouraged to gradually get sleepier. Time ratios regarding emblems of inspiration to expiration and mean values along with rate standard deviations in respiration cycled were averaged from the signals. We applied the Svm and KNN classifiers for detecting when an individual was drowsy or asleep. The proposed method was utilized alongside the Observer Rating of Drowsiness approach to grade the sleepiness levels of individuals. The k-fold and confusion matrix cross validation approaches were evaluated to understand better the classifiers and predictions regarding napping. These techniques gave an accuracy of up to 90% in detecting drowsiness, 92% in capturing the soundness, 85% in identifying the more specific explains, and 91% in well calculated measurement fulfilling the requirements of this study.

In^23^ authors provide a novel approach to the issue of driver drowsiness while behind the steering wheel using a system that is able to accurately identify drowsiness. In this system, Convolutional Neural Networks (CNN) are utilized in real time to ensure a higher degree accuracy while detecting the tendency of a driver to fall asleep. To classify the status of the eye, three networks were proposed, one of which is a Fully Designed Neural Network (FD-NN), and the other two are TL-VGG, which is solely Transfer Learning with VGG16 and VGG19 and has additional layers fused with them.

The authors in^24^ put forward the proposal of creating a system of real-time monitoring such as a webcam that detects drowsiness without pores physically interfacing the eyes such as an electrooculogram while ML and computer vision, which are more cost-effective, were employed. Automated alertness monitoring is essential for reducing the chances of human error, and the methodology being proposed employs drowsiness rules based on blink patterns sourced from neuroscience literature, this approach in neuro-physics, The Model exhibited tolerable behavior by not issuing alarms when participants were awake, and significantly flared up by issuing an average of 16.1 alarms for drowsy subjects, thus achieving an impressive accuracy of 94.44%.

The authors have developed a set of learning techniques that help in recognizing that a particular driver is drowsy and which are discussed in^10^. The deep learning models integrated into the system include but are not limited to: VGG-FaceNet, FlowImageNet, ResNet and AlexNet, where their primary function is analyzing video feed of the driver in order to ascertain if he is exhibiting the necessary signs. The entire system relies on a range of nodal features, head movements, gestures, behavior and facial expressions. For instance, AlexNet was integrated specifically for focusing fitting on sight ranges like indoor as well as outdoor. VGG-FaceNet transmits gender or ethnicity-related features while FlowImageNet interprets behavioral patterns and headturns, and ResNet discriminates hand motions. The enhanced hand motions recognition is a crucial aspect of the total system performance. These structures divide the recognized features into four groups: active normal state, yawning with blinking of eyes, clenching counterpart teeth, and nodding. With a real-time accuracy of 85%, the system is effective in detecting drowsiness in real time.

The authors in^25^ suggest a vision-based solution that applies an ensemble of two lightweight CNNs, each of them being responsible for a video stream and trained with eye patches. To mitigate the problem of overfitting when fine-tuning,” the aforementioned approach employs transfer learning. The system combines both; CNNs and fine-tune them in tandem to enhance the accuracy. The results of the experiments suggest that the proposed system, which the authors refer to as DCNNE, has a higher recognition success rate. For example, its accuracy on the ZJU dataset was 97.99%, which was more than the previous high of 97.20%. The model also performed well on the CEW, MRL and other databases. Moreover, the fine-tuned CNNs were implemented on two separate embedded devices assuring real-world application within time requirements.

According to^26^, a vehicle that protects itself from sleep-driving has been developed by the authors of the paper. The primary aim of their initiative is to protect individuals on the road from sleep-related accidents. Capturing these snapshots of driver’s face took no more than sixty seconds. Combined communication, behavior and recurrent networks were the first step towards reducing false rates. An additional fuzzy logic-derived approach allowed for manipulating visual data into up-to-date particulars about the driver.

The authors in^27^ develop an approach to image processing based detection of drowsiness recognition and traffic signs using the convolutional neural network, CNN. Accurate and efficient detection of road signs is essential in improving safety in road driving. Traffic signs contain essential data about traffic regulations, the state of the road, and intended travel for passengers, motorists, and pedestrians alike. To ignore or to circumvent traffic signs for any basis constitutes a high hazard for all road users and this project is a means to assist motorists to drive more responsibly.

The approach adopted in^13^ which involves a blend of deep learning techniques and CNN accompanied LSTM networks is novel as it predicts a driver’s drowsiness. CNN specializes in feature representation, and LSTM specializes in feature sequence construction which together, amplify the forecasting performance of the model. The model was given the NTHUDDD driver drowsiness dataset for testing and its performance was then registered against a range of old and modern systems. The evidence showed that the suggested model gave better performance with training accuracy reaching up to 98.30% and validation accuracy reaching to 97.31%.

In^11^, the authors proposed a model to estimate level of fatigue with eye movement analysis by a convolutional neural network. They also applied the CNN and VGG16 models for detecting and classifying the facial sleepiness poses into four states: open, closed, yawning and not yawning. Evaluation was carried out on a dataset of 2900 images of eye conditions due to boxer driver drowsiness, with gender, age, and brightness range included. For the scores displaying results, the levels of accuracy were also elevated with further assessments, the CNN model in this case received a 97 rating for the accuracy section 99 precision, recall and F-Score values, and 99 for the other sets. Notably, the VGG16 model achieved the converse 74% accuracy.

In^28^, the authors outlined a concept of the eye state classification based on convolutional neural networks, or CNN master adaptive mapping, and presented to it three simple CNN models, VGG16, VGG19 and new 4D models, for testing and sources. The MRL Eye dataset was utilized to train the 4D model, which was built specifically for eye condition assessment of sleepiness in the more advanced, quite darkened room. The 4D model also surpassed the pivot VGG16 and VGG19 models, having higher impressive state-predicting accuracy than the VGG16 and VGG19 models. This analysis presents an all-encompassing system of a driving model which predicts the state of the driver’s eyes in order to check if the person is feeling drowsy and sends alerts to improve safety on the roads.

In^29^, the authors suggested a non-invasive device for real-time monitoring of driver drowsiness that employs visual data obtained from car dashboard videos. The system uses facial landmarks and face mesh detectors to locate mouth aspect ratio, eye aspect ratio, and head pose, among other key regions. These features are used in three classifiers: random forest, sequential neural network, and linear support vector machine. The evaluation on the datasets from National Tsing Hua University showed that the system is capable of detecting drowsy drivers and sending appropriate alerts in time, with an accuracy of nearly 99%.

^30^, the authors have integrated deep learning technology in identifying driver fatigue with the aim of averting accidents. In their study they have detailed in their model training and testing Seven types of transfer learning based deep learning techniques which include VGG19, ResNet50V2, MobileNetV2, Xception, InceptionV3, DenseNet169, InceptionResNetV2 deep networks. Such models were analyzed and compared using the NTHU-DDD2 data set. Depicted as the most successful model reinforcing the deep learning models, VGG19 was noted to be very efficient. With the accuracy of 96.51%, precision 98.14%, recall of 95.36%, and a F1 score of 96.73%.

Authors in^31^ Study of a non-invasive drowsiness detection system operating in real time, which employs the use of convolutional neural networks, was thorough, detailed and demanding. Employing the technology One C and state-of-the-art video monitoring devices in the car cabin, the system analyses facial images emphasizing ascertaining whether the individual yawns and whether their eyes blink. A large training data set was used in the development of the system which encompassed images captured in an environment with different lighting conditions as well as taken from various angles of the face. Additionally, it makes use of Haar cascade classifiers for face region detection and more efficient methods of image analysis for determination of fatigue. The results of the experiment proved that the performance in the test reached 96.54% which evidenced how efficient behavioral indicators like yawns and eye state could improve image analysis process performance.

The authors in^18^ performed a detailed study on various drowsiness detection techniques with special emphasis on convolutional neural networks (CNN) along with transfer learning. In addition to their theoretical research, they also developed a relatively easy-to-use mobile application that utilizes these technologies. Many datasets were utilized to properly evaluate the developed model, establishing its efficacy. The drowsiness detection system as proposed was accurately between 90% - 99.86% for both multi and two-class detection systems.

The authors in^32^ proposed a powerful data-driven framework to capture drunk drivers’ behavior using t-distributed stochastic neighbor embedding (t-SNE) in conjunction with the Isolation Forest (iF) algorithm, where the authors autonomously confirmed the driver’s drunkenness level. As a feature extracting algorithm, the t-SNE model was chosen due to its property of maintaining local and global structures of feature space while performing dimensionality reduction on a nonlinear dataset. After feature extraction, the iF method unsupervised and tree-based anomaly detection model was applied, which has previously been trained to recognize only normal driving data, validating its use in practical scenarios. This study made use of publicly accessible sensor data from gas and temperature sensors and a digital camera, claiming an AUC of almost 95% and therefore high detection accuracy. The suggested approach also surpassed other techniques and combinations of dimensionality reduction and anomaly detection that included PCA, IPCA, ICA, kPCA, MDS with iForest, EE, and LOF, proving its strength and reliability in detecting alcohol-impaired driving behavior.

The authors in^33^ also integrated Independent Component Analysis (ICA), Kantorovitch Distance (KD), and the double Exponentially Weighted Moving Average (DEWMA) to propose a semi-supervised anomaly detection technique to classify and detect drunk driving behavior. In this approach modeling framework, ICA served the purpose of feature extraction for non-Gaussian multivariate data, while KD calculated the distance between normal and abnormal event intervals. DEWMA worked on the KD statistic to ensure sensitive change detection without a priori assumptions about the data distribution. To improve sensitivity, a nonparametric threshold was applied. This approach is particularly advantageous in real-world contexts, as labeled instances of drunk driving are hard to come by, since the method functions without labeled data. The study also XGBoost and used SHAP values to explain and defend feature importance in the detection process and interpretation. Evaluation done on a public dataset obtained from gas and temperature sensors and a digital camera showcased the model’s robustness, where it achieved an F1-score of 98%, significantly surpassing the performance of PCA and ICA based methods.

The prior research presented here has made remarkable advances in the drowsiness detection field by implementing real-time classification of the eye state. While most studies, such as those focused on CNN-based architectures or ensembles like the Dual CNN Ensemble (DCNNE), tend to accomplish a fair degree of accuracy, they struggle with generalizability and high computational costs. Moreover, SVM and KNN certainly have their merits; however, when stacked against deep learning models, they lack accuracy. Many models do not perform well across diverse datasets, or they are over-reliant on specific conditions, such as controlled lighting, for the environment in which they were trained.

The studies cited above serve as the baseline from which I propose my work leveraging the Vision Transformer (ViT) and Swin Transformer due to the self-attention-based feature extraction that aids those models in surpassing performance expectations. Those models have the ability to attend to precise features, unlike CNNs which tend to overlook important details in complicated real world scenarios. Moreover, the results that these transformer models offered, in my case high accuracy, especially using the ViT and Swin Transformer, shift the criticisms that were placed on the previous approaches based on CNN structures in terms of accuracy, robustness, and adaptability to varying conditions.

As shown in Table 2, previous studies have made substantial progress in driver drowsiness detection using CNN-based models and multimodal techniques. These approaches often demonstrate high accuracy when evaluated on constrained datasets; however, they tend to struggle with generalization in real-world conditions that involve variations in lighting, occlusion, head pose, and diverse facial features. In contrast, transformer-based models - although more computationally intensive - have recently shown promise in capturing long-range spatial dependencies and improving robustness. Despite their potential, few studies have applied transformer architectures specifically to driver drowsiness detection or incorporated interpretability mechanisms such as Class Activation Mapping (CAM) to enhance decision transparency. Table 2 provides a comparative summary of these related studies, outlining their datasets, techniques, accuracy, strengths, and weaknesses.

**Table 2:** The summary of the analyzed related works.

Ref	Year	Dataset	Classification model	Accuracy	Strengths and Weaknesses
^19^	2019	Self-prepared dataset	RF and non-linear SVM	RF: 88.37% to 91.18%	Strengths: Good performance with varying epoch lengths. Weaknesses: SVM accuracy is consistently lower
^20^	2019	CelebA, YawDD	Multiple CNN-kernelized correlation filters method	92%	Strengths: High accuracy in a variety of conditions, robust to environmental variations. Weaknesses: Limited to CNN models, lacks broader evaluation across other architectures
^21^	2020	300-W dataset	Mamdani fuzzy inference system	95.5%	Strengths: Incorporates fuzzy logic for drowsiness detection, useful for real-time applications. Weaknesses: May struggle with fine-tuning or handling complex image data without enhancement
^22^	2020	Self-prepared thermal	SVM and KNN	SVM: 90%	Strengths: Non-invasive method, useful for thermal monitoring in different lighting conditions. Weaknesses: Thermal imaging may require high-end equipment, and KNN struggles in complex environments.
		image dataset		KNN:83%	
^23^	2020	Self-prepared ZJU dataset	FD-NN, TL-VGG16, and TL-VGG19	FD-NN: 98.15%, TL-VGG16: 95.45%, TL-VGG19: 95%	Strengths: Impressive performance for fatigue detection, able to capture fine-grain eye movement features. Weaknesses: Limited dataset, reliance on predefined classifiers
^24^	2020	Self-prepared dataset (DROZY database)	Multilayer perceptron, RF, and SVM	SVM: 94.9%	Strengths: Good feature extraction using basic neural networks for fatigue detection. Weaknesses: Lack of deep learning-based approaches, might miss subtle facial cues.
^10^	2021	NTHU-DDD video dataset	Deep-CNN-based ensemble	85%	Strengths: Ensemble learning improves detection, high accuracy in diverse environments. Weaknesses: Lower overall performance compared to transformer-based models
^25^	2022	CEW, ZJU, MRL	Dual CNN Ensemble (DCNNE)	CEW: 97.56%, ZJU: 97.99%, MRL: 98.98%	Strengths: High performance across multiple datasets with ensemble methods. Weaknesses: May not generalize well to datasets outside of the tested range
^26^	2022	UTA-RLDD dataset	RNN and CNN	60%	Strengths: Low computational cost, suitable for mobile applications. Weaknesses: Low accuracy, particularly for complex real-time applications
^27^	2022	Self-prepared dataset for traffic signs	CNN	98.53%	Strengths: Strong accuracy in traffic-related scenarios, applicable to many monitoring systems. Weaknesses: Lacks focus on drowsiness detection, limited to specific contexts
^13^	2022	NTHU-DDD	CNN + LSTM	97.3%	Strengths: Efficient for sequential data analysis, effective in dynamic environments. Weaknesses: Struggles with long-duration analysis or sustained predictions in real-time
^32^	2022	Public dataset using gas sensor, temperature sensor, and digital camera	t-SNE for feature extraction + Isolation Forest (iF) for anomaly detection	95%	Strengths: Effective detection using only normal (non-drunk) data, Handles nonlinear, high-dimensional data well, Unsupervised approach suitable for real-time detection. Weaknesses: t-SNE is computationally intensive and not ideal for real-time processing, Model performance may vary with different sensor quality or configurations
^11^	2023	Drowsiness dataset	CNN and VGG16	CNN: 97%, VGG16: 94%	Strengths: High performance in detecting drowsiness from real-time data. Weaknesses: Limited to VGG16 architecture, potential underperformance in new data types
^28^	2023	MRL	VGG16, VGG19, and 4D	VGG16: 95.93%, VGG19: 95.03%, 4D: 97.53%	Strengths: Strong results across multiple configurations, robust for real-time driver drowsiness detection. Weaknesses: Dependence on specific VGG-based models may limit flexibility in dynamic environments
^29^	2023	NTHUDDD dataset	RF, SVM, and sequential NN	RF: 99%, SVM: 80%, 4D: 96%	Strengths: RF offers excellent performance, especially for simple fatigue detection scenarios. Weaknesses: SVM underperformed significantly, less robust across diverse environmental conditions
^33^	2024	Public dataset using gas sensor, temperature sensor, and digital camera	ICA for feature extraction + Kantorovitch Distance (KD) + DEWMA for anomaly detection; XGBoost for SHAP analysis	F1-score = 98%	Strengths: Does not require labeled data (semi-supervised) High sensitivity using DEWMA with nonparametric threshold, SHAP adds explainability to the model, Effective on non-Gaussian multivariate data. Weaknesses: Complexity due to integration of multiple techniques, DEWMA and KD may require careful tuning for different datasets, Potentially computationally intensive for real-time systems
^30^	2024	NTHU-DDD	VGG19	96.51%	Strengths: Efficient in various lighting and environmental conditions. Weaknesses: Performance variation across datasets, still limited by fixed network architectures
^31^	2024	YawDD, MRL	VGG16 and CNN	VGG16: 95.85%, CNN: 96.54%	Strengths: High performance in real-time detection with various feature extraction methods. Weaknesses: Not ideal for low-complexity devices, might need more robust processing power
^18^	2024	MRL	CNN, InceptionV3, and MobileNetV2	CNN: 96%, MobileNetV2: 97%, InceptionV3: 98%	Strengths: Excellent performance with quick response times, particularly in driver monitoring. Weaknesses: InceptionV3 and MobileNetV2 still face computational trade-offs

## Proposed methodology

**Fig. 1:**
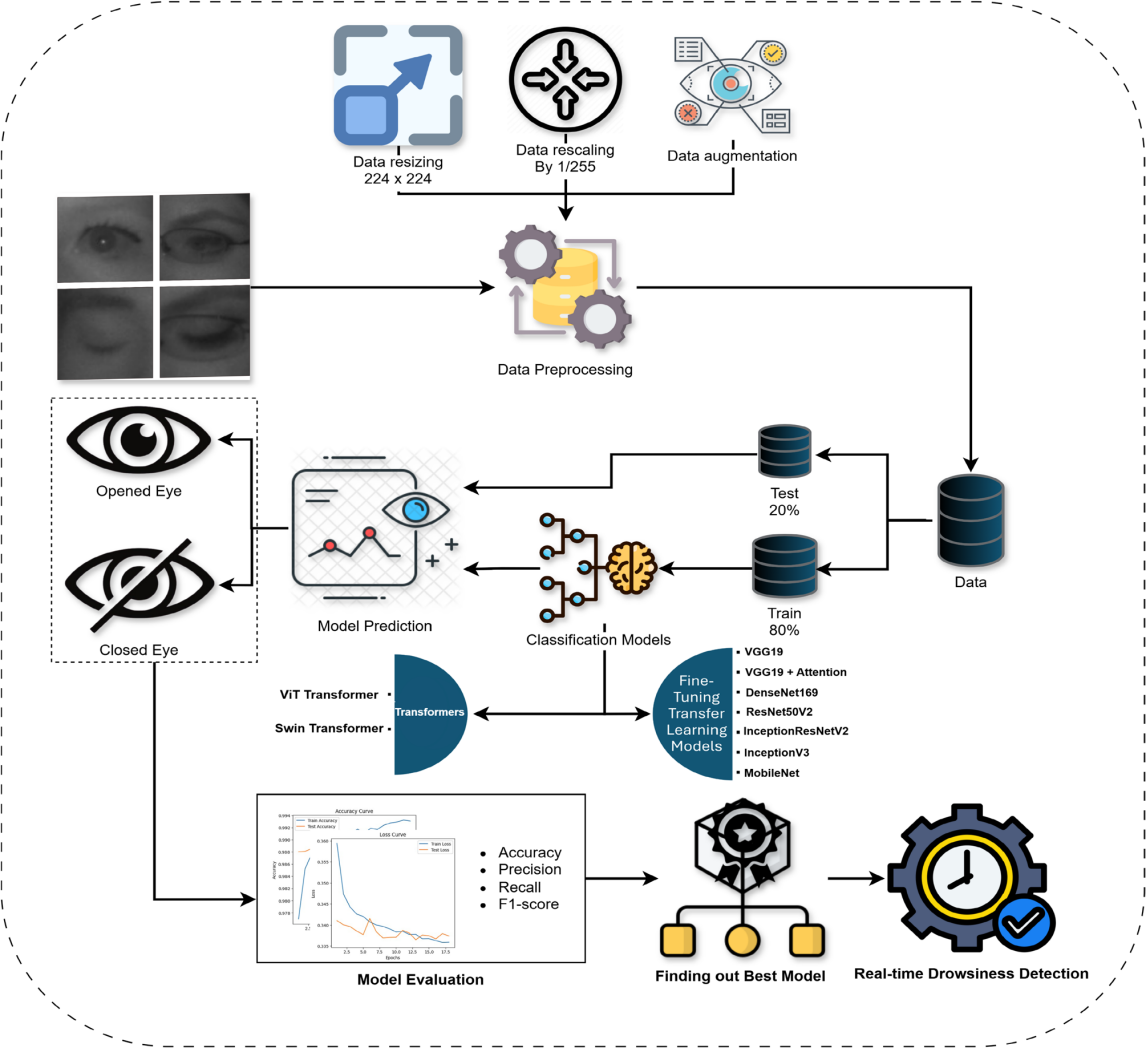
The workflow architecture.

This paper introduces a comprehensive and systematic methodology for real-time driver drowsiness detection, leveraging state-of-the-art deep learning techniques to classify eye states into “Open-Eyes” and “Close-Eyes.” The proposed framework is designed to address the limitations of existing approaches by integrating advanced data preprocessing, transformer-based architectures, and transfer learning models. The methodology begins with data preparation, including image resizing, normalization, and augmentation, to enhance the generalization capabilities of the models. The MRL, NTHU-DDD, CEW datasets are utilized, with an 80-20 split for training and testing, ensuring a robust evaluation of the models. A diverse set of deep learning architectures, including Vision Transformer (ViT), Swin Transformer, and fine-tuned transfer learning models such as VGG19, DenseNet169, ResNet50V2, InceptionResNetV2, InceptionV3, and MobileNet, are trained and evaluated based on key performance metrics such as accuracy, precision, recall, and F1-score. The best-performing model is then deployed in a real-time system that utilizes Haar Cascade classifiers for face and eye detection, coupled with a drowsiness scoring mechanism to trigger alarms when prolonged eye closure is detected. This end-to-end approach not only ensures high accuracy but also provides a practical and scalable solution for improving road safety.The summary diagram of how the driver drowsiness detection model works is shown in Fig. 1 which outlines the sequence of steps followed in this research.

**Fig. 2:**
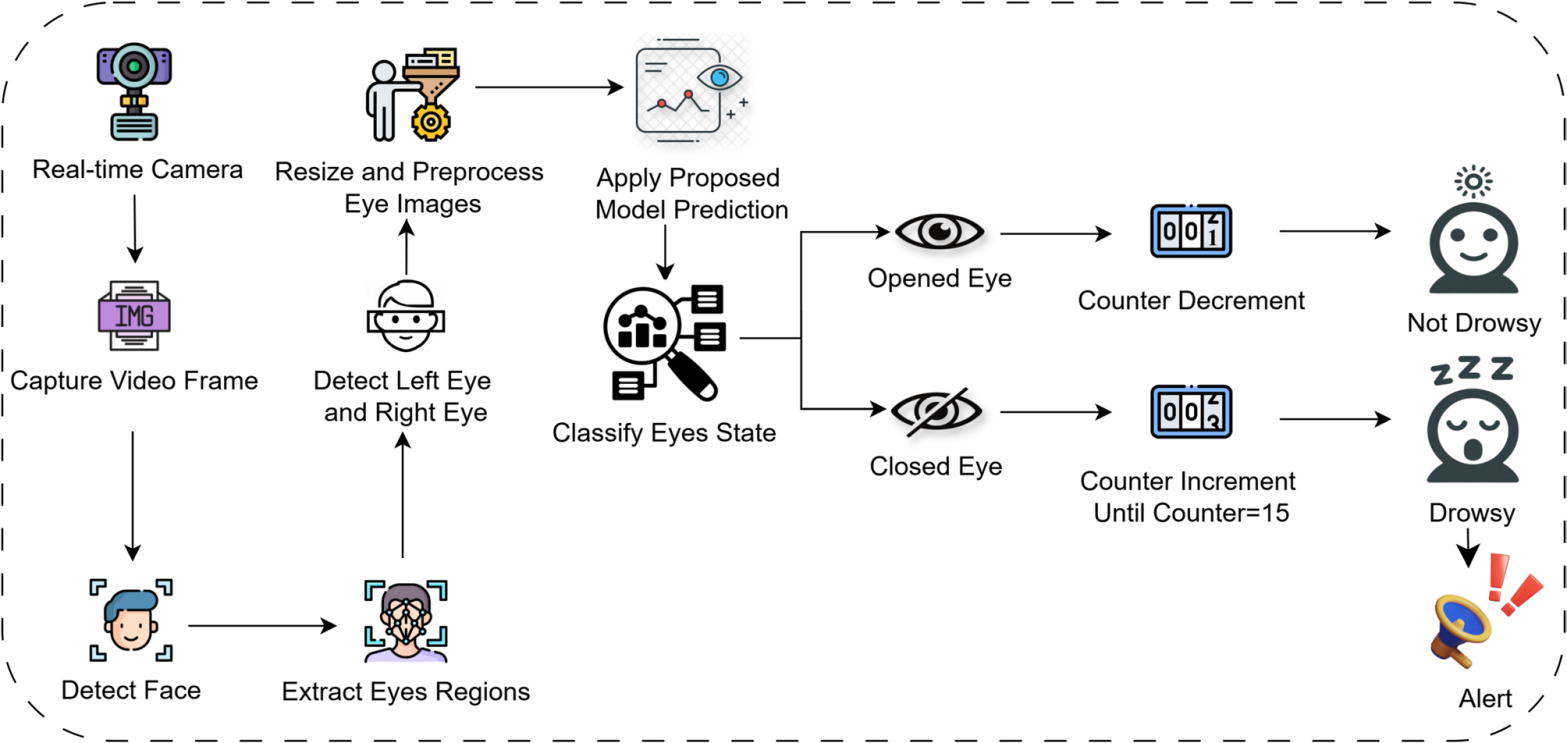
Real-time detection architecture.

For the purpose of evaluating drowsiness, videos are processed by detecting the frames in real time and then classifying them in real time, starting from driver’s face to both the left and right eye using Haar Cascade classifiers. The captured eye regions of the driver are preprocessed to conform to the set standards of the model including scaling as well as normalization, once this step has been completed, the model with optimally set parameters derived from the previous workflow is deployed to identify whether the eyes were open or closed. Every drowsiness score starts from zero, a score increases by 1 unit in case closed eyes are detected. The score for drowsiness will be triggered in the case that the score has remained constant for 15 frames, meaning the driver will alert once a frame reaches a certain threshold. If the predictions generated indicate the eyes of the driver are open, then the score is decreased by one. The real-time detection process is visually represented in Fig. 2.

### Dataset description

With emerging technologies, selecting appropriate datasets in the field of driver drowsiness detection is crucial, as they are deemed fit for robust model calibration as well as generalizability. Every dataset comes with its own set of challenges affecting the system’s detection accuracy and performance. These hurdles include variations in lighting, facial occlusions from glasses or other objects, head movements, and subtle but impactful slow blinking and yawning, as well as other sleepy behaviors. Understanding these dataset-specific issues is essential for model development, preprocessing strategies, and evaluation. The key challenges associated with the NTHU-DDD, CEW, and MRL datasets used in this study are summarized in Table 3.

**Table 3:** Key challenges in drowsiness detection datasets.

Dataset	Key challenges
MRL	Poor lighting conditions in night mode
	Presence of eyeglasses
	Close-up eye images only (no full face)
	Grayscale images only
NTHU-DDD	Varying facial angles and head pose
	Different lighting conditions (day/night)
	Slow blink with nodding
	Yawning
	Sleepy combinations
	Eye occlusion due to glasses
CEW	Varying head orientation
	Wide diversity in facial expressions
	Presence of eyeglasses and sunglasses
	Occlusions and real-world variability (e.g., hair, hand)

For the experiment, the MRL Eye Dataset^28^ is used. This dataset has been popular for other research as well notably for drowsy driver detection tasks that focus on eye-state recognition. The MRL dataset contains 84,898 samples in total, all of which can be classified into two main categories, these being Open-Eyes and Close-Eyes. In the aforementioned categories there are 42,952 images for Open-Eyes and 41,946 images for Close-Eyes; Hence, the two categories are distributed almost equally. The images included in this dataset span different resolutions, light conditions, and even different orientation of the eye: so it is a very hard dataset for construction of effective classification models. All these factors suit our research - the dataset’s size allows for deep learning models that are able to detect drowsiness in real-life cases.

NTHU-DDD dataset is a comprehensive and diverse collection of driver videos recorded in real vehicles under daytime and nighttime environments. It has annotated behaviors such as yawning, slow blinking, and nodding, along with sleepy states’ combination, recorded at various facial angles and head movements. This dataset helped us significantly in our work by enabling us to test a complete range of sleepy behavior and account for performance in operating conditions that are representative of real-world scenarios. Within the dataset as a whole, there are 66521 640 $\times$ 480-resolution grayscale images of each of the two classes, i.e., drowsy and not-drowsy. There are 36030 sleepy images in the dataset altogether and 30491 images altogether which are not sleepy.

Closed Eyes in the Wild (CEW) dataset contains a collection of facial images with closed and open eyes that were collected in unconstrained scenarios. It presents huge variation in facial orientation, expression, and occlusions such as glasses and hair. CEW was employed in our research as a cross-validation reference to measure model generalization to in-the-wild data, simulating real-world deployment scenarios where changing visual features should be correctly understood. The CEW dataset comprises 27200 images of closed and open eyes. In contrast to the MRL dataset, the CEW dataset comprises full-face images, posing additional challenges in the guise of occlusions, different lighting, head pose variation, and motion blur. These conditions make the dataset very representative of real-world driving situations, where environmental conditions are changing and unpredictable.

**Fig. 3:**
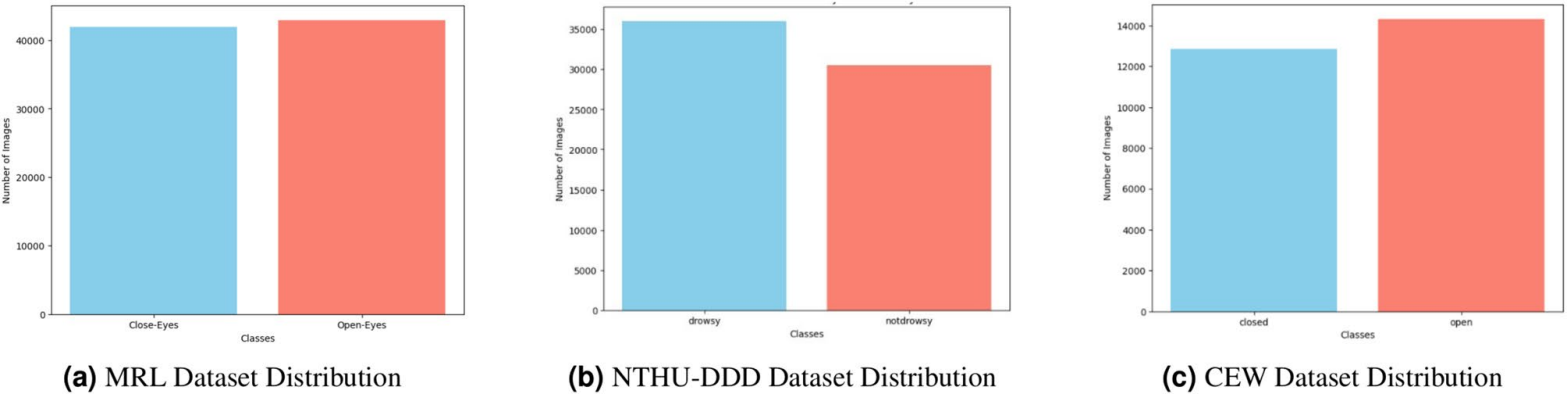
Dataset distribution across MRL, NTHU-DDD, and CEW.

**Fig. 4:**
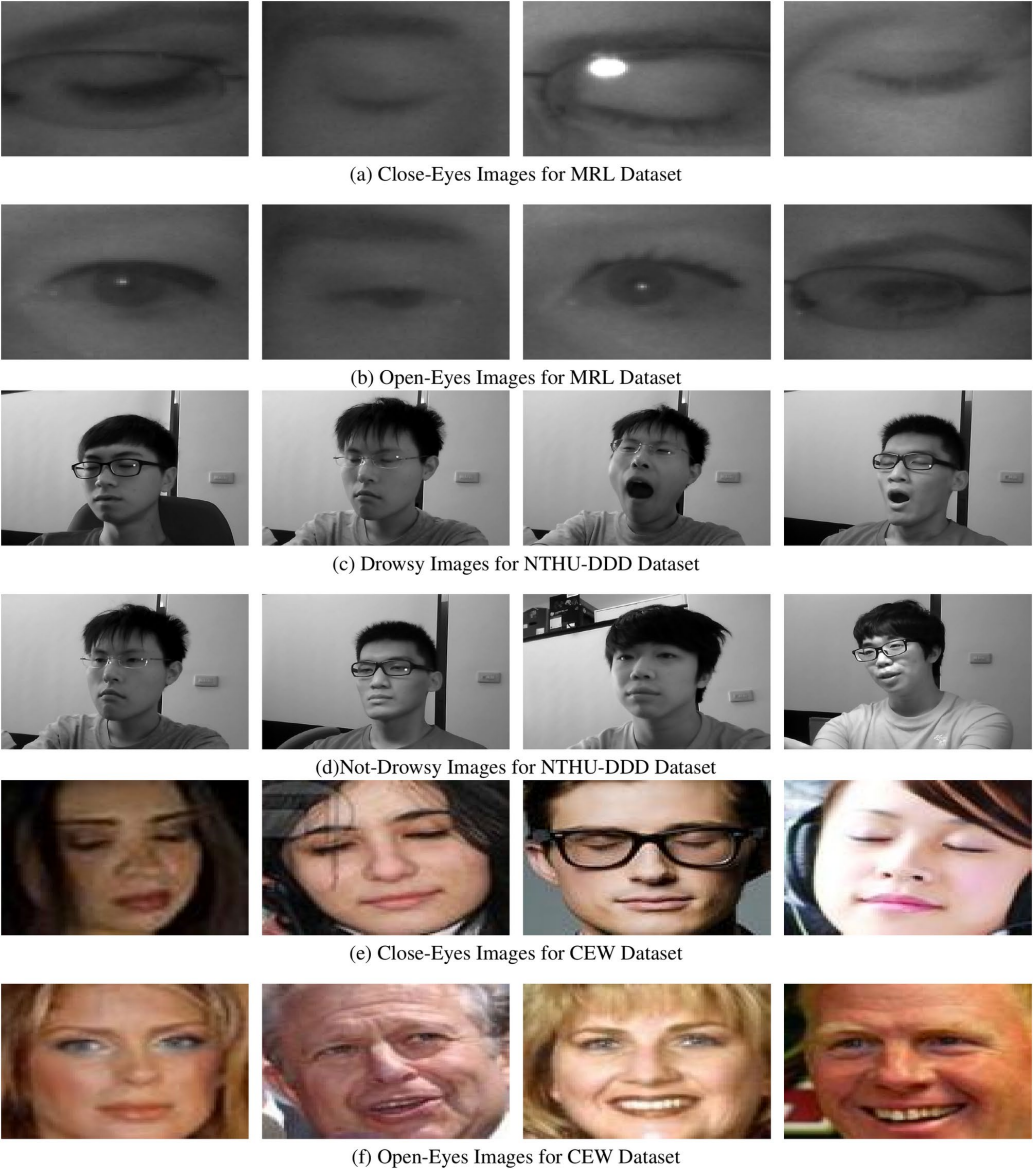
Sample images.

Interpreting Figs. 3 and 4 represent the data distribution, and sample images of the obtained images from the dataset. The dataset has a total of two purposes hence two classifications; one for training, and one for testing. In simpler terms, out of the total dataset 80% was allocated for training and 20% reserved for testing.

### Data preprocessing

This part introduces the need of data preprocessing. This is one of the core processes in both machine learning and deep learning as it minimizes the chances of over fitting and generalizes the model. Any kind of working on the input data to make it ready for further action is termed as “data preprocessing.”. The concept of preprocessing in data makes a lot of applications such as machine learning, deep learning, data mining, and computer vision more friendly and effective. For the purpose of this experiment, we carry out data normalization and scaling. It is necessary to ensure that all images are of the same size in order to allow for the same dimensional input throughout the model. In our experimental of the proposed model, all the images were scaled to dimensions of $224 \times 224$. The model learning process is improved by reducing the influence of external pixel values. To attain that, each pixel is normalized into the range of [0,1] by dividing each pixel value by 255.

In an effort to prevent overfitting and make the model more generalizable, data augmentation methods are utilized on the MRL dataset throughout the preprocessing phase. Many transformations are done on the training images to keep their main characteristic intact while being changed. The ShiftScaleRotate tools in this case moves random images off center and out of proportion, the model then grows stronger to these changes in the position of the eye. HorizontalFlip is often paired with images to give a reflection to the rest of them to strengthen and prepare the model for different angles. Images RandomBrightnessContrast changes the brightness and contrast of images allowing the model to work under different lighting scenarios. Some more tools are rotation_range, width_shift_range, height_shift_range that slightly rotate and move images vertically and horizontally in order to simulate real life movement of the head and the position of the eyes. As a result of editing with these augmentation tools the model became stronger at identifying Open-Eyes and Close-Eyes states giving better real-time drowsiness detection.

### Region of interest selection

This study uses Haar cascade classifiers to detect, locate and extract the face and eye regions for drowsiness detection preprocessing. Haar cascade is a technique based on machine learning, where essential features are gained from a portrait, or integral image, and categorized utilizing AdaBoost^34^. The algorithm scans an image over different intervals, and identifies elements depending on pre-instructed features. The classifier scans parts of an image over different intervals, employing predefined features. In this case, face or eyes, it seeks special features such as the bridge of the nose is lighter than the eyes, the eyes are darker than the cheeks and the forehead is lighter than the eyes. Because of the classification algorithm, face is extracted and from the extracted face box, eye regions are extracted. This step is performed to reduce probabilistic and computational cost of false positives.

**Fig. 5:**
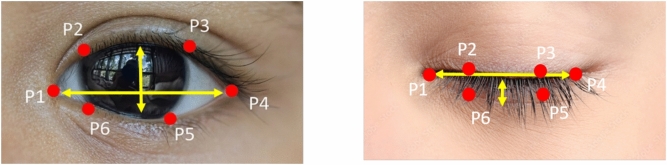
Eye extraction.

The shape of a human eye can be described by a set of points. The relative position of these points defines how open or closed the eye is. So as to measure how open an eye is, it’s essential to know which 2D landmarks to use around the eyes^35^. There are some important points p1 to p6 are the 2D facial landmarks of the eye as shown in Fig. 5. The eye’s state, open or close, is detected by calculating the Eye Aspect Ratio (EAR) as shown in Eq. (1). The vertical distances between the eye landmarks are summed up and placed in the numerator, whereas those placed horizontally are in the denominator. A lower EAR value refers to a closed eye, while a higher value refers to an open eye. An EAR score less than 0.25 would define the eye as closed whereas an EAR that scores above will define the eye as open.

1 $$\begin{aligned} EAR = \frac{\Vert P_2 - P_6 \Vert + \Vert P_3 - P_5 \Vert }{2 \times \Vert P_1 - P_4 \Vert } \end{aligned}$$

After detecting the face, the Haar cascade classifier is trained to detect the eye region, after which a deep learning classifier is used to classify the extracted eye images. The model predicts the unlocked or locked status of the eye, which adds to the overall drowsiness score. If the score from both eyes is under a certain value for a set time, then an alarm is sounded. This combination of Haar cascade classifier for Region of Interest (ROI) selection and deep learning for classification proved to be effective and accurate for real-time drowsiness detection.

### Ethical statement

The human face visible in Fig. 27 is that of one of the authors. The author has provided informed consent for the use of their image in this manuscript. All methods and procedures involving human subjects were carried out in accordance with relevant guidelines and regulations. No institutional or licensing committee approval was required for the use of the author’s image, as it was voluntarily provided for research purposes.

### Transformer architectures

The advancement of transformers in deep learning particularly natural language processing and image processing models cannot be ignored. They were first introduced in the research paper ‘Attention Is All You Need’^36^ and in this paper, the architecture has replaced the fully connected recurrent and convolution structures by self attention which allows models to be able to tackle full sequences or full images effortlessly.

With those properties, transformers are suitable in image processing tasks considering the fact that they can be used to model complex dependencies between far apart elements in space. Not like CNNs that are built upon local areas, vision transformers in a similar fashion to traditional ones, self attention is spread all over the parts of the images which make them outperform other network models on numerous tasks such as image identification and locating objects within an image.

Meanwhile, the Swin Transformer Adds ons to ViT by proposing a window shifting approach alongside a multi level bidirectional design to enable quick processing and enhance the efficacy of working with large images. Vision transformer’s issues are addressed in the Swin Transformer by using a sliding window approach and also resulted in maintaining a productive result in vision activities while also lessening computation cost.

#### ViT transformer

ViT is among the first transformer-based models used to perform image classification tasks^37^. While CNNs rely on convolutional layers to hierarchically analyze the features of images, ViTs process images by dividing them into fixed-size patches which get linearly embedded and treated as a sequence through the use of a Transformer encoder^38^.

ViT has a number of primary elements: The Classification Head is the last part to process the feature mapping to enable an image determined to be Open-Eyes or Close-Eyes when combined into a fully connected layer. The Transformer decoder, which comprises a number of self-attention layers and feed forward networks that function alongside the patch embeddings. ViT also has a classification kernel, or Layer Merge. Embedded images have positional information pasted on them, typically 16x16 patches. The self and cross attention modules together ensure that the spatial data learned is used. ViT has proven useful on large scale datasets especially for image classification tasks^12^. In this case, ViT is slightly altered and instead of determining whether a driver is awake or not, it determines whether a drivers eyes are open or closed or in other words whether a driver is.

The MRL dataset fine-tuning starts with resizing images adjusted to 224$\times$224 pixels. Also, horizontal flipping is used at random to promote better training. The first step of training starts with a weight trained model google/vit-base-patch16-224 which is adjusted for a two state eye model and an AdamW optimizer is applied with a rate of 5e-5 with a CrossEntropyLoss applied. The model is equipped with an early stopping validator with a patience of five. For the computing conditions, the model doesn’t start depending on a bias since, the masks aren’t utilized, achieving balanced multi orientated images, however the model utilizes a default value of 32 across the line up of 30 and splitting testing and training. Performance was judged by metrics of loss and accuracy during the two sets of training and testing that were held in a single session. The process is stopped as soon as one of the early stopping parameters is reached to ensure that the model doesn’t overfit and handles testing well.

**Fig. 6:**
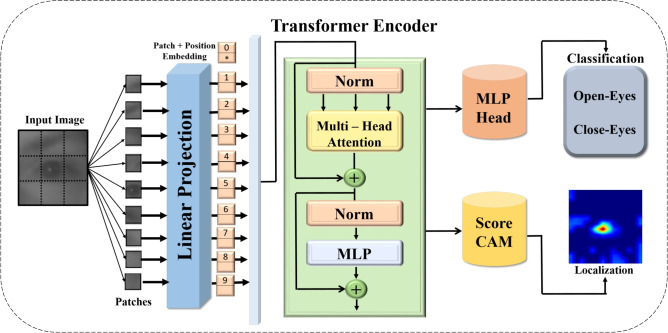
The ViT transformer architecture.

The proposed Vision Transformer (ViT) model that has been submitted for drowsiness detection works by splitting input images into smaller patches followed by a linear projection accompanied by the addition of positional embeddings. These linear projections ensure that spatial information is preserved. The patches that have been transformed are then run through a number of transformer encoder layers. Here, global dependencies are captured by mechanisms of multi-head self-attention along with normalisation layers which improve the model’s robustness. The model also includes a Head, MLP, and Multi-Layer Perceptron for classification into Open-Eyes and close-Eyes states. More so, Class Activation Mapping (CAM) is implemented for enhanced visibility/easier understanding of the decision made by focus modeling and at the same time identifying clearly the areas that were of interest in the decision. The block diagram of this structure is presented in Fig. 6. It visually depicts the different stages in the process of extracting and classifying features.

#### Swin transformer

The Swin Transformer architecture involves multiple components. To begin with, the image is sliced into square blocks with no overlaps and then a Patch Embedding Layer is used to map the image blocks into higher dimensional vectors. The structure of the Swin Transformer is hierarchical in nature and it enables local and global dependencies to be captured via the use of shifting windows of self-attention^39^. The self-attention blocks are restricted within each window and once again the windows are moved throughout the layers of the self attention model so that the model can learn cross-window interactions. In addition, patch merging layers are employed in order to down sample the feature map thereby permitting the model to learn deeper scales features. At last, a classifier head is utilized to make the final decision and classifies the grayscale image as Open-Eyes or Close-Eyes. The Swin Transformer does well in image classification problems dealing with high-resolution images^40^ and is tuned in this work to drowsiness detection of a person’s eyes whether open or closed.

We take an image with the label of Open-Eyes or Close-Eyes and send it through our model trained specifically from the Hugging Face SwinForImageClassification swin-tiny-patch4-window7-224 instance. To achieve a binary classification, the model undergoes further adjustments. The image encoder is swapped with a custom one that has a dropout layer and a linear projection layer which are used to tackle overfitting. Razoring images with random flips and alterations to contrast and brightness up prepares the dataset for the model enlargement allowing the model to deal with a larger variety of classes. The AdamW optimizer is combined with a learning rate and a cross-entropy loss function to train the model, streamlining the fine-tuning procedure. Other than that, early stopping is used while monitoring the validation loss to ensure the model trains efficiently without overfitting. Moving weight averages (SWA) are applied to boost generalization and improve overall performance. After the model is fine-tuned to our needs, we conduct further evaluations using accuracy, loss metrics and drowsiness detection optimizations allowing the model to distinguish between Open-Eyes and Close-Eyes efficiently.

**Fig. 7:**
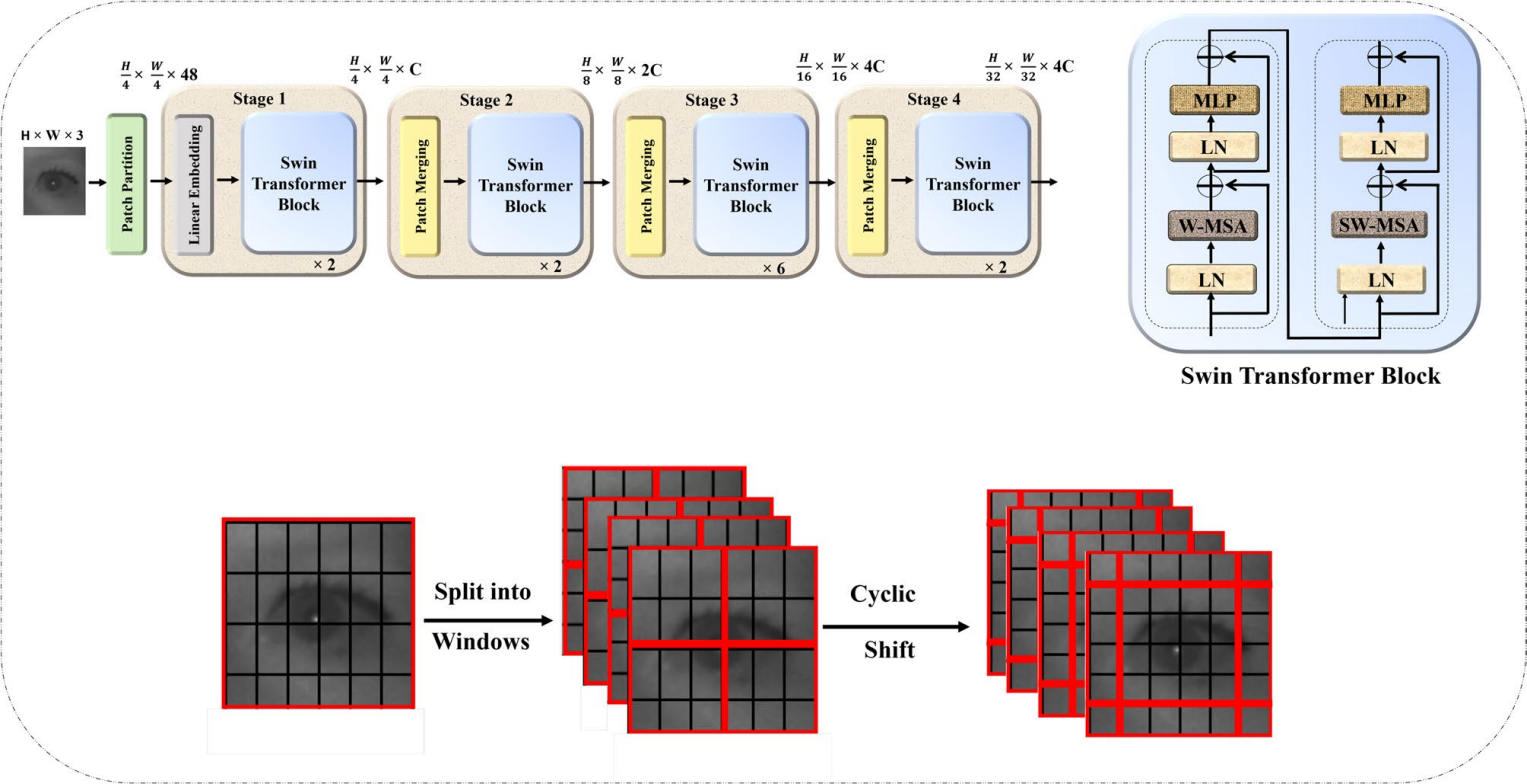
The swin transformer architecture.

To ensure maximum effectiveness, a set of hyperparameters are established in the Swin Transformer model during the fine tuning process. The model is set to a batch size of 32, this helps in effective image data processing during the training and evaluation phases. Different learning rates are applied to the backbone and classifier parts of the model: 1e-5 is defined in the former and 1e-3 is defined in the latter. As a result in the issued setting, the parameters learn the last classification layers faster than 1e-5. The training is set to span across 30 epochs and has an early stopping mechanism which halts the training process after 5 epochs without an improvement in validation loss. This is done to mitigate the risk of overfitting. A weight decay of 1e-4 is set in the loss function to facilitate the parameters optimization process using AdamW optimizer. Label Smoothing Cross Entropy is the loss function applied with a smoothing factor of 0.1, this reduces both overfitting and increases generalization. The block diagram of the Swin transformer is presented in Fig. 7. It visually depicts the different stages in the process of extracting and classifying features.

### CNN models architectures (fine-tuning transfer learning models)

#### VGG19

The visual geometry group at Oxford University introduced the VGG architecture that is extended into what is now known as VGG19^41^. It was developed in the year 2014 and consists of Nineteen layers, out of these layers—out of which 16 are convolutional and 3 are fully connected, along with that it contains strategically placed max-pooling layers which serve to reduce the spatial dimension while retaining important features. Just like the VGG16 model, the VGG19 model also uses 2 $\times$ 2 max-pooling kernels and 3 $\times$ 3 convolutional filters, they enhance the efficiency of the entire design of the system. With the addition of the layers, VGG19 has enhanced capacity to perform deep feature extraction, hence allowing it to perform complex image classification with high accuracy.

Since the VGG19 is hierarchical in nature, it is able to recognize intricate patterns with ease^42^. The very basic and simple features such as edges and textures are concentrated in the shallow layers however more higher level features, like facial movements or jaw movements, are contained within the deeper sides of the layer. The fine granularity that this allows is useful in scenarios where driver drowsiness detection is needed and xeyelid closure and prolonged blinking are informative. Althought the architecture cost for the VGG19 is higher than that of the VGG16, the accuracy and robustness in the detection of fatigued drivers is better in VGG19.

The VGG19 model has a keen aptitude when it comes to telling distinguishing features of a face that’s associated with drowsiness, especially when it is used with well trained domain centered datasets like MRL. Sitting down for this further improves its performance at being able to tell the difference between open and closed eyes which works well with letting it being used in systems that are aimed for monitoring the state of a driver in real time. With fatigue based patterns being effectively determined along with the high-level accuracy that the model provides, it makes it great for use in various situations while a person is behind the wheel.

#### VGG19 + attention

In this subsection, we describe the process of fine-tuning a pre-trained VGG19 model with an attention mechanism to classify the open-eye and close-eye states within the MRL dataset. The aim is to make use of the feature extraction of VGG19 but at the same time, disentangle with attention block over relevant image features.

The model of the fine-tuned VGG19 is composed of the already trained VGG19 model but without the fully connected layers on top, preloaded with weights obtained from ImageNet. We then freeze the first 15 layers to keep the pre-trained feature extraction and allow the rest of the deeper layers to be trained on the dataset. A multi-layer perceptron attention mechanism is used to enhance the feature maps by first applying global average pooling, dense layers and a sigmoid activation function to the input feature maps. Global Average Pooling eliminates unnecessary spatial information but retains the major residual components. Fully connected layers of 512, and 256 neurons were then added with ReLU activation and with dropout for regularization. The last layer is a softmax classifier that predicts two output classes: Open-Eyes and Close-Eyes.

Now, let’s analyze the channel attention mechanism that is able to weight feature maps given by VGG19. It first applies Global Average Pooling which converts the feature maps to a single channel. Then, the vector is processed with fully connected layers that are aimed at preserving some essential spatial relationships. A sigmoid function is the last step, it generates attention weights that are then multiplied with feature maps over individual elements so that important areas can be highlighted^43^.

To enhance the performance of the model while testing, data augmentation is applied to the training images, which include a degree of random rotations, width and height shifts, as well as, horizontal flipping. Both the training and test data is obtained from folders and scaled to 224 $\times$ 224. Since this is a multi-class classification problem, the categorical class mode is used.

In order to increase performance, a few measures have been taken. The training is stopped after a predetermined number of epochs without improvement of the validation loss. This is done to avoid overfitting. In addition, there is a model checkpoint that saves the model with the best validation accuracy in order to restore the appropriate weights later. If the validation loss does not improve, the learning rate is lowered then, which helps in achieving convergence.

After the model has been trained, it goes through an evaluation stage where it is provided with a test dataset. In this stage, a few key metrics, such as the accuracy of the model and the loss are noted and recorded. The particular model that has the lowest loss is saved and used for classifying eye states of the drivers in real time to assess their drowsiness in future applications.

#### DenseNet169

DenseNet169 is a deep convolutional network that is about 169 layers deep and was developed to eliminate the vanishing gradient problem^44^. The goal was to greatly increase the effectiveness of learning new features while decreasing usage of parameters. It is efficient when it comes to leveraging previous knowledge, and while doing so, facilitates the propagation of gradients. Each layer was designed to pull information from all layers at once after itself, in doing so, it was able to enhance the redundancy of parameters.

DenseNet169 perceives intricate multi-level features that are essential for recognizing driver drowsiness, specifically for drivers who have some signs of closed eyes and slow blinking, as they are slight eye movement indicators, this proves useful to wide driver fatigue detection^45^.

Constantly adjusting the DenseNet169 on the MRL dataset increases the effectiveness with which the model can tell supporting it with the capability of classifying the model accurately. Dense connectivity architecture broadly assists with the capability of generalization ensuring vast and immense performance of the model in real life driver monitoring situations.

#### ResNet50V2

ResNet50V2 is a Microsoft Research based deep convolution neural network with residual learning which is an updated version of ResNet50^46^. This network consist of fifty layers which include identity and convolutional residual blocks which enhance the propagation of gradients leading to resolving the problem of vanishing gradients. Because of this architecture, networks can be deeper while maintaining stability during training, making it efficient in tasks that require complex image classification.

Driver drowsiness detection gets a huge boost from ResNet50V2’s deep hierarchical feature extraction capabilities^47^. The initial layers focus on basic image properties while the high level ones zoom eye closing and minute facial expressions. And as the model relies on residual connections, that ensures there will be greater efficiency with avoiding overfitting during fine tuning the model to the MRL data set.

The alter as described above allows ResNet50V2 to perform better with MRL dataset as it can now effectively discriminate Open vs Closed eyes and thus classify drowsiness states. The model does not suffer from any generalization issues suggesting it’s viable for real world deployment where the use case is driver supervision in real time, ensuring the detection is accurate regardless of the light or surrounding.

#### InceptionResNetV2

InceptionResNetV2 combines Inception and ResNet into a single framework as it adds inception modules with residual connections to boost accuracy and loss to gain efficiency^48^. Because of this hybrid design, there is less need to worry about the complications that arise when training a model when the networks are very deep since there is a high degree of accuracy achieved.

Because of its features, the model is appropriate for driver drowsiness detection. Its inception blocks are residual, and they can identify complex and abstract facial features which are relevant such as the closure of one’s eyes and other signs of fatigue^30^.

By applying InceptionResNetV2 on a particular dataset called MRL, we can improve the model’s ability to effectively distinguish between closed and opened eyes. A real time system that monitors a driver to determine if they are drowsy can be possible while ensuring safety on the roads against accidents that are caused by fatigue, owing to InceptionResNetV2, which is able to extract relevant features and learn efficiently.

#### InceptionV3

InceptionV3 is another great product that Google developed. It is an AI algorithm that makes use of deep learning and specializes in Amalgamation neural networking processes for efficient computational resource and cost reduction of an enhanced deep learning model. Two other of its components are classifier aids and de-factored filtering^49^.

Inception V3 also allows monitoring of drowsy drivers as it is able to capture fine and coarse details which will construct proper facial features. When coupled with inception modules, it becomes easier to precisely identify eye closure and other signs relating to fatigue which improves the necessary cue in the monitoring process to control the drowsiness of the driver.

To a large extent, the reliability of the driver is enhanced such that InceptionV3 has been fine-tuned on the MRL dataset greatly improving its monitoring tools and detecting drowsiness for drivers regardless of their status. Being safe, the software continues to prove extremely efficient in real-time applications.

#### MobileNet

MobileNetV2 is a light-weight Convolutional Neural Network specifically targeted for mobile and embedded systems. It was developed by Google and employs the two techniques of depthwise separable convolutions and the inverted residual blocks^50^. These techniques decrease the complexity of computations without losing a lot of accuracy. Owing to its efficient architecture, MobileNetV2 is well-suited for use in environments with limited resources and which require real-time inferences.

The capability of MobileNetV2 to hierarchically extract features, makes this architecture appropriate for detection of driver drowsiness. This model is competent in determining facial indicators like eye closure and extended blinking of the eyes; which is not computationally intensive at all. This efficacy particularly helps in embedded applications using real-time analytics, where every millisecond counts.

MobileNetV2’s eye classification is fine-tuned on the MRL dataset which allows for the quick determination of either open or closed eyes and thus drowsiness is detected with low latency. Furthermore, this architecture is easily implemented on in-vehicle driver monitoring systems and thus ensures that timely warnings about fatigue related issues are issued. The problem with this model is that it gives low accuracy.

## Experiments and results

### Experimental setup

The ViT transformer model and eight different models (Swin Transformer, VGG19 with Attention, VGG19, DenseNet169, ResNet50V2, InceptionResNetV2, InceptionV3, and MobileNet), all of those are implemented in Python using the Kaggle platform. The execution of the code happened against the following system configuration, as represented in Table 4

**Table 4:** System configuration details.

Configuration	
IDE	Kaggle
Programming Language	Python
Libraries	Tensorflow, Keras, Torch, Pandas, Matplotlib, scikit-learn
GPU	NVIDIA Tesla P100 with 16 GB VRAM
CPU	Intel Xeon CPU (2.3 GHz, 46 MB cache)
RAM	16 GB of system memory

### Training phase

The images belonging to the MRL dataset are subdivided into two sets for the purposes of the training phase. The categorization of the dataset into two sets is found to be 80% training and 20% testing. Hence, the total number of images is 84,898, with 67,917 images used for training and 16,981 images used for testing. Similarly, the NTHU-DDD dataset is also divided using an 80%-20% train-test split, comprising a total of 66,521 images, of which 53,216 images are allocated for training and 13,305 images for testing. In addition, the CEW dataset follows the same train-test ratio, with a total of 27,200 images, including 21,760 training images and 5,440 testing images. This distribution is summarized in Table 5.

**Table 5:** Number of images in each dataset.

Categories	MRL dataset	NTHU-DDD dataset	CEW dataset
Total	84,898	66,521	27,200
Train	67,917	53,216	21,760
Test	16,981	13,305	5440

### Performance evaluation methods

The model performance was evaluated based on some common metrics like confusion matrix, accuracy, recall, precision, and F1 score.

#### Confusion matrix

The confusion matrix comprises a table of correct and wrong predictions made by the model. It is derived from four parameters: True Positive, True Negative, False Positive, and False Negative. The definitions of these parameters are as follows:

- (TP): correct prediction of class1 when the actual label is class1.
- (TN): correct prediction of class2 when the actual label is class2.
- (FP): prediction class1 when the actual label is class2.
- (FN): prediction class2 when the actual label is class1.

These four values from confusion matrix are utilized to calculate accuracy, precision, recall, and F1-score. These metrics provide a comprehensive evaluation of the model’s performance in classifying “Open-Eyes” and “Close-Eyes”.

#### Accuracy

It’s the number of instances that the classifier had predicted correctly divided by the total number of instances. Accuracy is nothing but the overall correct representation of the model. It is calculated using:

2 $$\begin{aligned} \text {Accuracy} = \frac{TP + TN}{TP + TN + FP + FN} \end{aligned}$$

#### Precision

Precision is the ratio of correctly predicted positive instances to the predicted positive instances. This is very useful when the cost of false positives is critical. Precision is calculated as follows:

3 $$\begin{aligned} \text {Precision} = \frac{TP}{TP + FP} \end{aligned}$$

#### Recall

Recall, also known as a sensitivity or truth positive rate, measures the ratio of actual positives that a model is able to capture through its prediction. Recall would be a scenario where we want to minimize false negatives. It is calculated using:

4 $$\begin{aligned} \text {Recall} = \frac{TP}{TP + FN} \end{aligned}$$

#### F1Score

This means that the F1 score takes into account both precision and recall, and is defined as the harmonic mean of precision and recall, thereby generating a single metric that captures the degree of trade-off between these two: Its definition is:

5 $$\begin{aligned} \text {F1-score} = \frac{2 * \text {Precision} * \text {Recall}}{\text {Precision} + \text {Recall}} \end{aligned}$$

### Hyperparameters

The selection of hyperparameters in this study was based on a combination of empirical testing and values commonly recommended in prior research. Initial values for learning rate, batch size, and number of epochs were adopted from similar deep learning applications in driver monitoring and visual classification tasks (e.g.,^51,52^). These values were then fine-tuned using manual experimentation on a validation split of the training data. For each model, early stopping was applied with a patience of 5 to prevent overfitting. While we did not employ exhaustive grid or random search due to computational constraints, iterative testing allowed us to achieve reliable convergence and performance across datasets.

Hyperparameter tuning is essential when it comes to deep architectural structures to achieve the optimum performance. Several of the hyperparameters used included batch size, learning rate, epochs, optimizer chosen, loss function used, early stopping on patience, and all of these were used during the training to improve the classification accuracy on the MRL data set.

- Batch size: The batch size is the number of training samples to work through before the internal model parameters are updated. A bigger batch size produces slower stable gradient updates but necessitates more memory; a smaller batch size results in more gradient updates and, whilst the training is noisier, convergence is quicker. After experimentation, a size of 32 batches was found to be ideal as it is a more cost-effective than advocating for model performance.
- Learning rate: The learning rate determines the size of each update step for weights in gradient descent. A model could take too long to converge if the learning rate is set too low, while a model could overshoot the optimal setting with a higher learning rate. For the purposes of this research, a learning rate of 0.0001 was used since this enabled reliable yet quick convergence during training.
- Number of epochs: The number of epochs limits the number of times the complete model will work with the training data. An increased number of epochs can cause the model to undergo overfitting, while a reduced number of epochs can induce underfitting. In conjunction with early stopping, 30 epochs were used at maximum in order to allow the model to end training early, should there be no observable performance improvement.
- Optimizer: The optimizer chosen does affect the rate of convergence as well as the extent to which convergence is achieved. For this paper, We have used the Adam Optimizer able to use the advantages of learning rates that self-adapt.
- Loss function: A binary cross-entropy loss function was utilized in training the model since this is suitable for binary class problems. Such functionality calculates the difference between the predicted values and the values represented by the given labels thereby helping the model decrease training error.
- Early stopping with patience: The method of early stopping was used with patience of 5 epochs, with an aim to reduce overfitting and increase performance on unseen datasets. It indicates when there is no enhancement in the validation loss for a span of 5 epochs, the training cycle needs to be terminated. The use of early stopping slightly resolved expending computational resources by not allowing training beyond its optimal point and was efficient in aiding in conflict of overfitting by securing an optimal stopping point.

Table 6 depicts the values for some hyperparameters in the experiment. Adjustment of these hyperparameters led to improved generalization on unseen data with no compromise on computational costs. The values chosen ensured that there was no fitting of the model on the data and there was likewise no overfitting to create a robust classification model that could successfully detect open and closed eyes in the MRL dataset classification model.

**Table 6:** Hyperparameter settings.

	Fine-tuning Transfer learning models	ViT transformer	Swin transformer
Batch size	32	32	32
Learning rate	1e-4	5e-5	Backbone: 1e-5 classifier: 1e-3
Epoch	30	30	30
Early stopping	Patience = 5	Patience = 5	Patience = 5
Optimizer	Adam	AdamW	AdamW (decay:1e-4)
Loss	Binary CrossEntropy	CrossEntropyLoss	Label smoothing cross entropy (0.1)

Table 7 presents the size and estimation of total parameters, trainable parameters, and others for each of the nine models. The complexity of each model can be seen in the table.

**Table 7:** Total parameters comparison of the models.

Sr. no.	Model name	Image size	Trainable parameters before fine-tuning	Trainable parameters after fine-tuning	Model size (MB)
1	ViT transformer	224 * 224	85,800,194	85,800,194	327.30
2	Swin transformer	224 * 224	27,914,108	27,914,108	106.48
3	VGG + attention	224 * 224	12,226,850	12,226,850	78.02
4	VGG19	224 * 224	2,099,201	143,668,241	548.05
5	DenseNet169	224 * 224	1,705,985	14,190,465	54.74
6	ResNet50V2	224 * 224	2,099,201	25,618,561	97.90
7	InceptionResNetV2	224 * 224	1,574,913	55,851,105	213.29
8	InceptionV3	224 * 224	2,099,201	23,867,553	91.18
9	MobileNet	224 * 224	1025	3,208,001	12.32

### Accuracy evalution

This research compares several sophisticated deep learning approaches, including Tensorflow models such as ViT Transformer, Swin Transformer, VGG19 with Attention, VGG19, DenseNet169, ResNet50V2, InceptionResNetV2, InceptionV3, and MobileNet against multiple images containing “Close-eyes” as well as “Open-eyes.” All of the models were subjected to early stopping with a patience value of 5 for a maximum of 30 epochs kept for training. The models’ performance was evaluated in terms of accuracy, precision, recall, and F1-score. The results for the MRL dataset are listed in Table 8, the NTHU-DDD dataset in Table 9, and the CEW dataset in Table 10. The MRL dataset served as the primary dataset for training and evaluating all models, while the NTHU-DDD and CEW datasets were employed to validate the generalizability and robustness of the proposed models under more diverse, real-world conditions. Notably, the Transformer-based models-specifically ViT and Swin-consistently outperformed all other models across the datasets, demonstrating superior accuracy compared to both traditional machine learning and transfer learning-based CNN architectures. To ensure the robustness and statistical reliability of the results, each experiment was conducted over five independent runs, with accuracy reported as a mean ± standard deviation. It was observed that classical machine learning models performed significantly weaker, especially when applied to complex and varied real-world image conditions. These findings further validate the advantage of Transformer architectures in capturing intricate spatial dependencies and enhancing model generalization in real-time driver drowsiness detection tasks.

**Table 8:** Performance evaluation and comparison of various models on the **MRL** dataset.

Model	Precision (in %)	Recall (in %)	F1 score (in %)	Accuracy (in %)
ViT Transformer	99	99	99	99.15 $(\pm 0.12$)
Swin Transformer	99	99	99	99.03 $(\pm 0.13$)
VGG19 + Attention	99	99	99	98.85 $(\pm 0.11$)
VGG19	98.5	98.5	99	98.7 $(\pm 0.15$)
DenseNet169	98.5	98.5	99	98.65 $(\pm 0.14$)
ResNet50V2	98.5	98.5	99	98.5 $(\pm 0.12$)
InceptionResNetV2	98.5	98.5	98.5	98.5 $(\pm 0.13$)
InceptionV3	98.5	98.5	98.5	98.5 $(\pm 0.14$)
MobileNet	98	98	98	97.99 $(\pm 0.11$)
Random Forest	98	98	98	97.93 $(\pm 0.11$)

**Table 9:** Performance evaluation and comparison of various models on the **NTHU-DDD** dataset.

Model	Precision (in %)	Recall (in %)	F1 Score (in %)	Accuracy (in %)
ViT Transformer	99	100	100	99.52 $(\pm 0.09$)
Swin Transformer	99	99	99	98.76 $(\pm 0.11$)
VGG19 + Attention	99.44	98.52	98.98	99.07 $(\pm 0.12$)
VGG19	98	98	98	98.66 $(\pm 0.13$)
DenseNet169	98.52	98.44	98.48	98.60 $(\pm 0.12$)
ResNet50V2	97.41	99.48	98.43	98.55 $(\pm 0.13$)
InceptionResNetV2	98.74	94.70	96.68	97.02 $(\pm 0.14$)
InceptionV3	98.71	97.74	98.22	98.38 $(\pm 0.13$)
MobileNet	90.20	89.52	89.86	91.15 $(\pm 0.12$)

**Table 10:** Performance evaluation and comparison of various models on the **CEW** dataset^53^.

Model	Precision (in %)	Recall (in %)	F1 score (in %)	Accuracy (in %)
Swin Transformer	100	100	100	100 $(\pm 0.0 )$
ViT Transformer	97	95	96	97.25 $(\pm 0.12 )$
VGG19 + Attention	97	97	97	98.17 $(\pm 0.11 )$
VGG19	96	93	95	96.3 $(\pm 0.12 )$
DenseNet169	95	95	95	96.3 $(\pm 0.14 )$
InceptionResNetV2	89	94	91	93.6 $(\pm 0.12 )$
MobileNet	91	90	91	93.6 $(\pm 0.13 )$
InceptionV3	88	89	88	91.7 $(\pm 0.13 )$
ResNet50V2	85	86	86	89.9 $(\pm 0.12 )$

#### ViT transformer

**Fig. 8:**
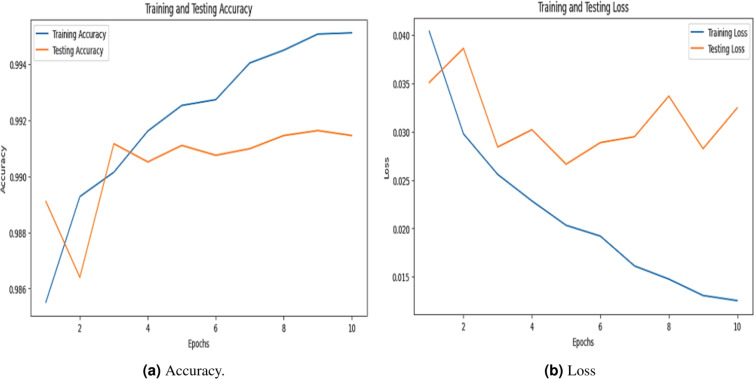
ViT transformer accuracy and loss graphs on testing MRL dataset.

**Fig. 9:**
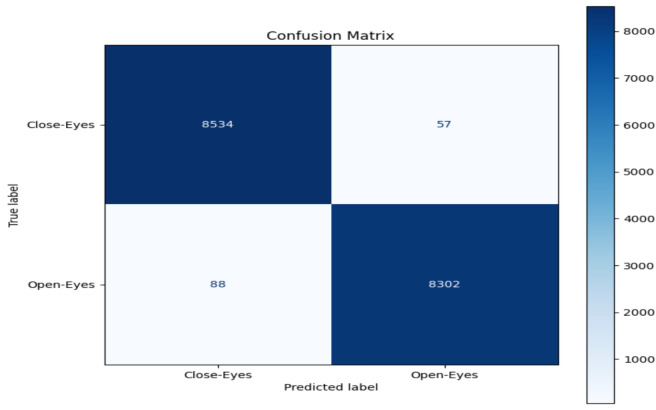
ViT transformer confusion matrix for MRL dataset.

The ViT Transformer performed remarkably well, earning the highest scores in every metric of the analysis. It achieved an accuracy rate of 99.15% and also impressive precision and recall of 99%. Moreover, the F1 score of the model is 99% which is a strong indication of its good balance between recall and precision. The accuracy plot in Fig. 8a illustrates a well-defined accuracy curve that indicates good learning stability, while Fig. 8b shows a low loss convergence trend demonstrating strong training dynamics. Furthermore, the confusion matrix which is presented in Fig. 9 contains a few misclassification errors which support the high accuracy and reliability of the model.

#### Swin transformer

**Fig. 10:**
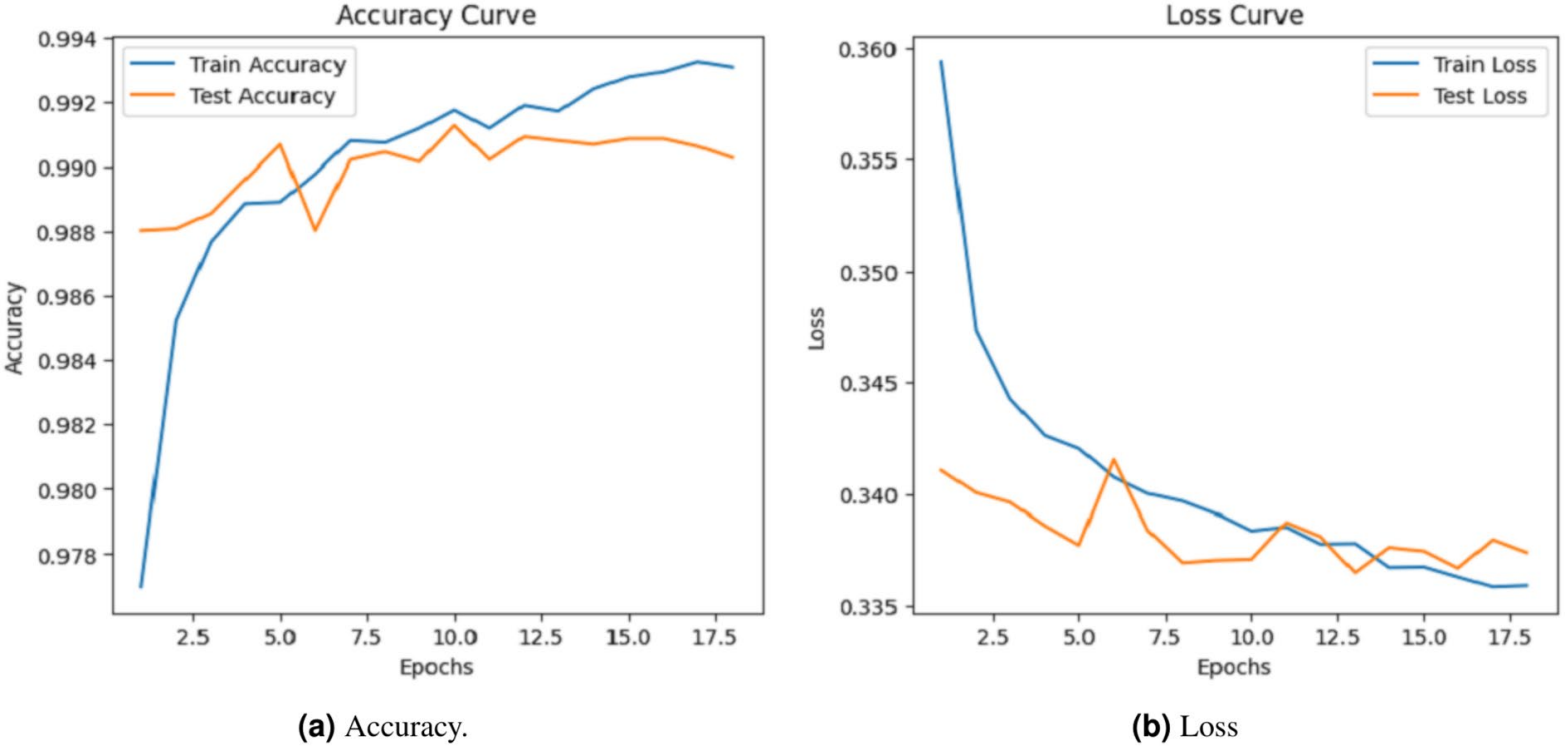
Swin transformer accuracy and loss graphs on testing MRL dataset.

**Fig. 11:**
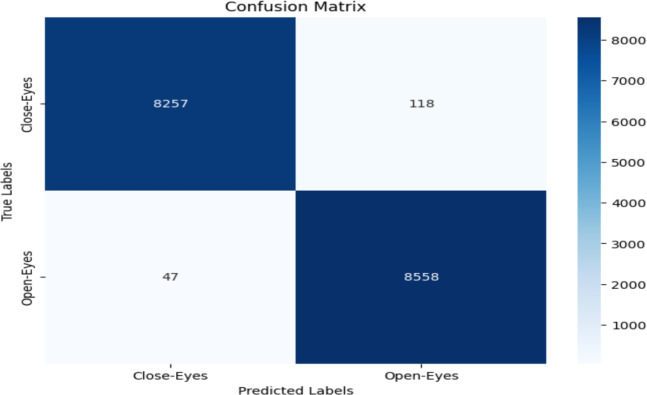
Swin transformer confusion matrix for MRL dataset.

The Swin Transformer model performed impressively closely to the ViT model amassing an impressive accuracy of 99.03%. It managed to maintain good performance across the board with all three metrics scoring 99% each for precision, recall and F1 score. The model performance improved steadily as shown in Fig. 10a . The loss curve in Fig. 10b also provides evidence of optimization of the model. Examining Fig. 11, the confusion matrix also illustrates the discriminative capacity of the model showing only a handful of misclassification.

#### VGG19 + attention

**Fig. 12:**
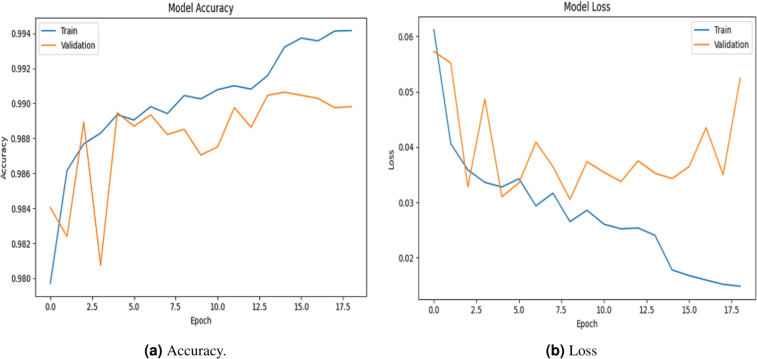
VGG19 with attention accuracy and loss graphs on testing MRL dataset.

**Fig. 13:**
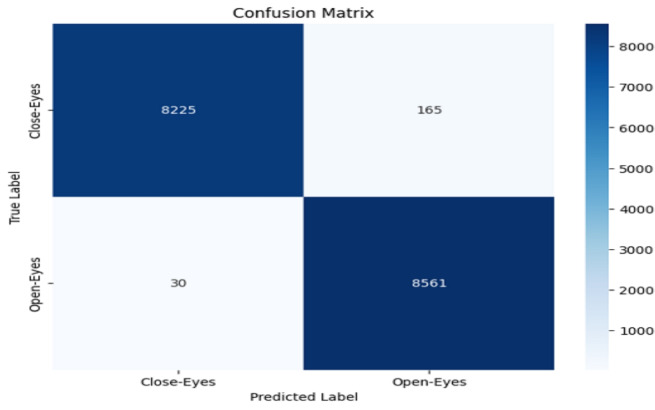
VGG19 with attention confusion matrix for MRL dataset.

VGG19 with the Attention mechanism achieved slightly lower results than the ViT Transformer and Swin Transformer with an accuracy of 98.85%. The model had 99% precision and recall resulting in the F1 score of 99%. The accuracy progress presented in Fig. 12a shows good learning stability, which was also complemented by the loss curve in Fig. 12b pointing to strong convergence. The confusion matrix in Fig. 13 further illustrates the strong performance of the model in the classification of different categories.

#### VGG19

**Fig. 14:**
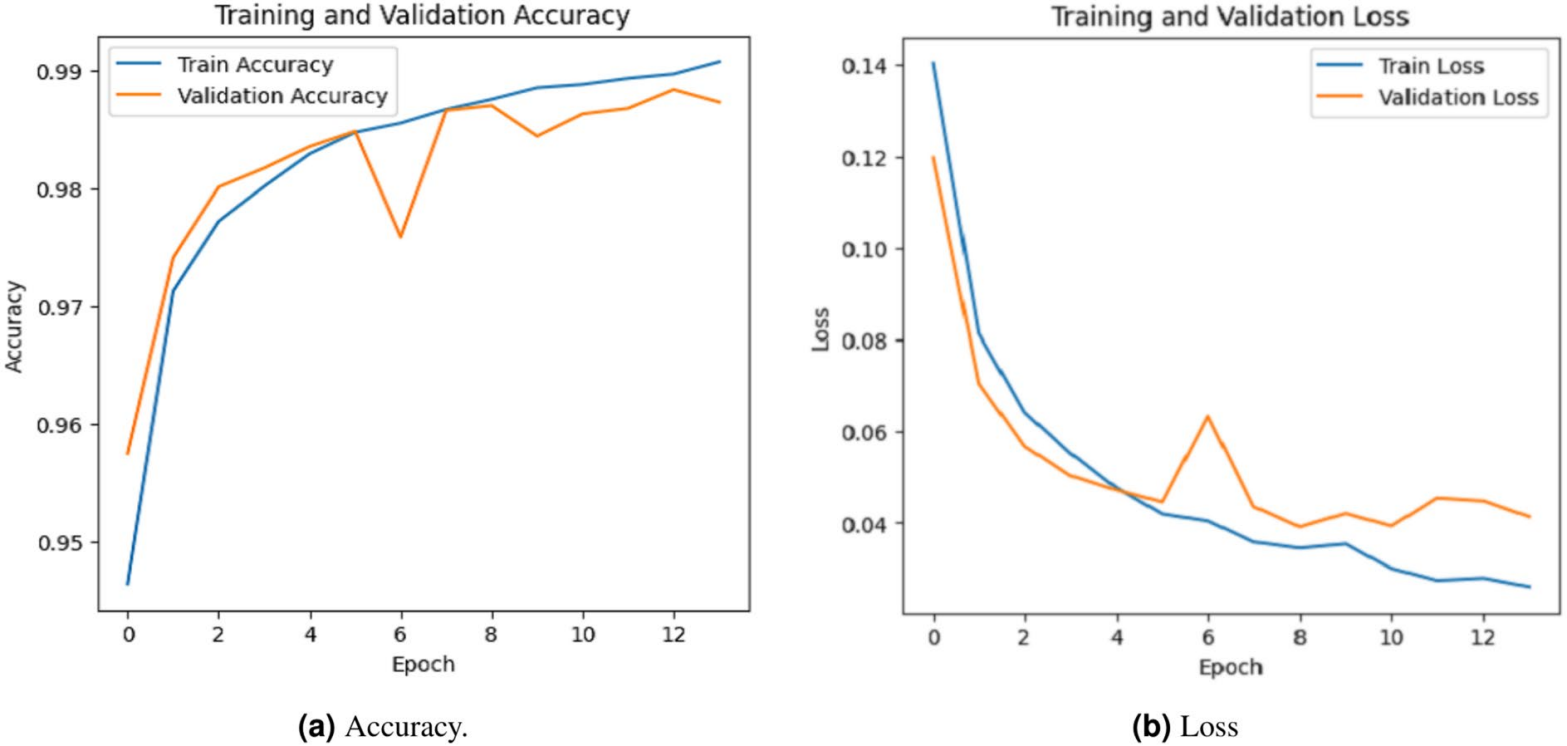
VGG19 accuracy and loss graphs on testing MRL dataset.

**Fig. 15:**
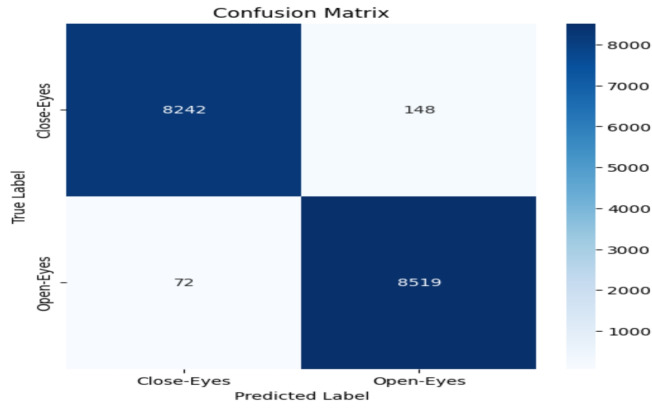
VGG19 confusion matrix for MRL dataset.

The measurements gathered indicated that VGG19 model produced an accuracy of 98.7%, recall of 98.5%, F1 score of 98.5% and a precision of 98.5%. As it can be seen in Fig. 14a , the accuracy curve indicates that graph learning remained smooth for multiple epochs. From Fig. 14b we see a loss curve where the model appeared to converge, though more gradually as compared to the other models. Lastly, the confusion matrix is represented in Fig. 15.

.

#### DenseNet169

**Fig. 16:**
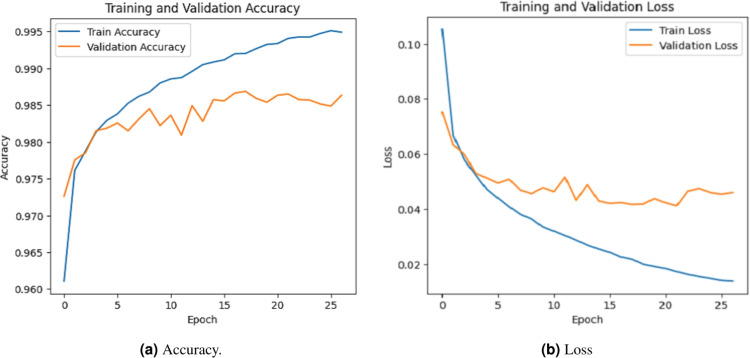
DenseNet169 accuracy and loss graphs on testing MRL dataset.

**Fig. 17:**
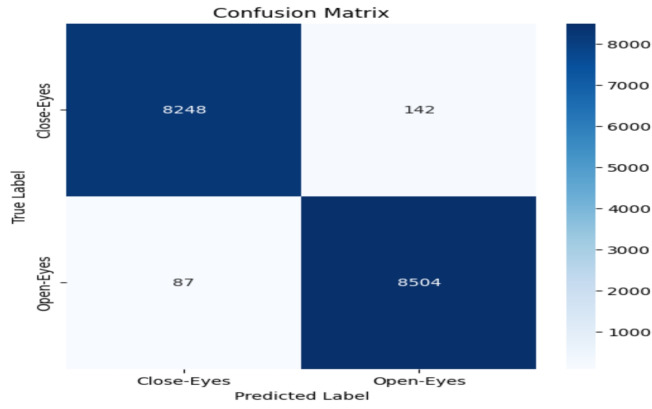
DenseNet169 confusion matrix for MRL dataset.

DenseNet169 model, on the other hand, achieved an accuracy of 98.65% with other parameters-precision, recall, and F1 score of 98.5%. In Fig. 16a the accuracy curve maintains a proper equilibrium suggesting step learning while the loss curve as seen in Fig. 16b displays strong convergence with very low overfitting. Referring to Fig. 17, the confusion matrix depicts a modest misclassification rate compared to the best models but overall maintains a high degree of perceived reliability.

.

#### ResNet50V2

**Fig. 18:**
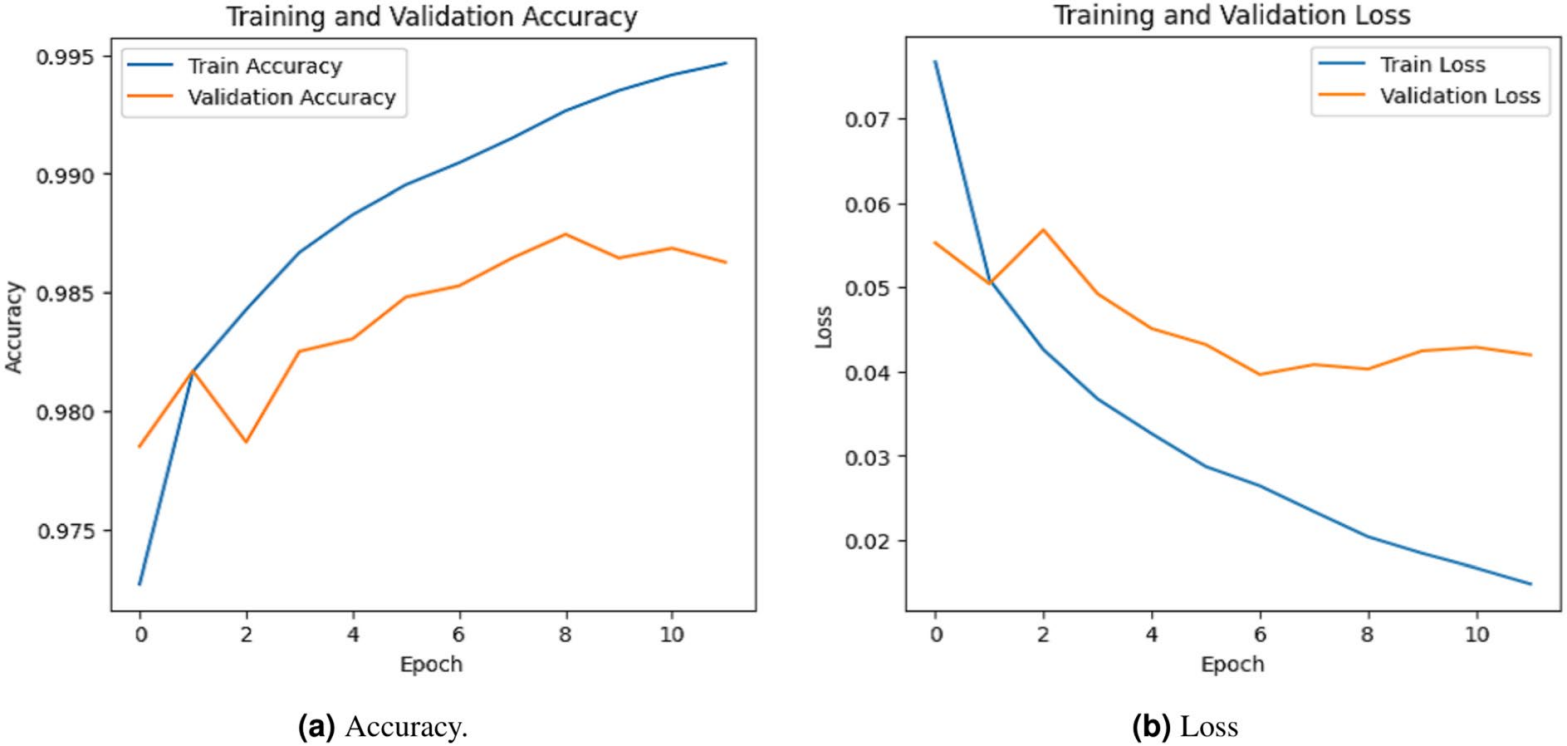
ResNet50V2 accuracy and loss graphs on testing MRL dataset.

**Fig. 19:**
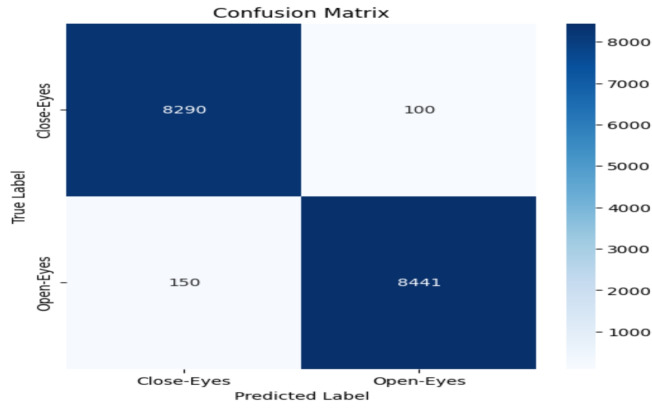
ResNet50V2 confusion matrix for MRL dataset.

The ResNet50V2 model performed exceptionally well, achieving an overall accuracy of 98.5% along with F1 score of 99%, recall of 98.5% and precision of 98.5%. The accuracy curve as depicted by Fig. 18a managed to grow exponentially which leads to the model’s improvement across various epochs. Furthermore, Fig. 18b showcases that the ResNet50V2 had a low loss curve while being trained. Similarly, the figure addressed above features the confusion matrix which shows that the ResNet50V2 performed just well enough as the DenseNet169 by avoiding misclassification as shown in Fig. 19.

.

#### InceptionResNetV2

**Fig. 20:**
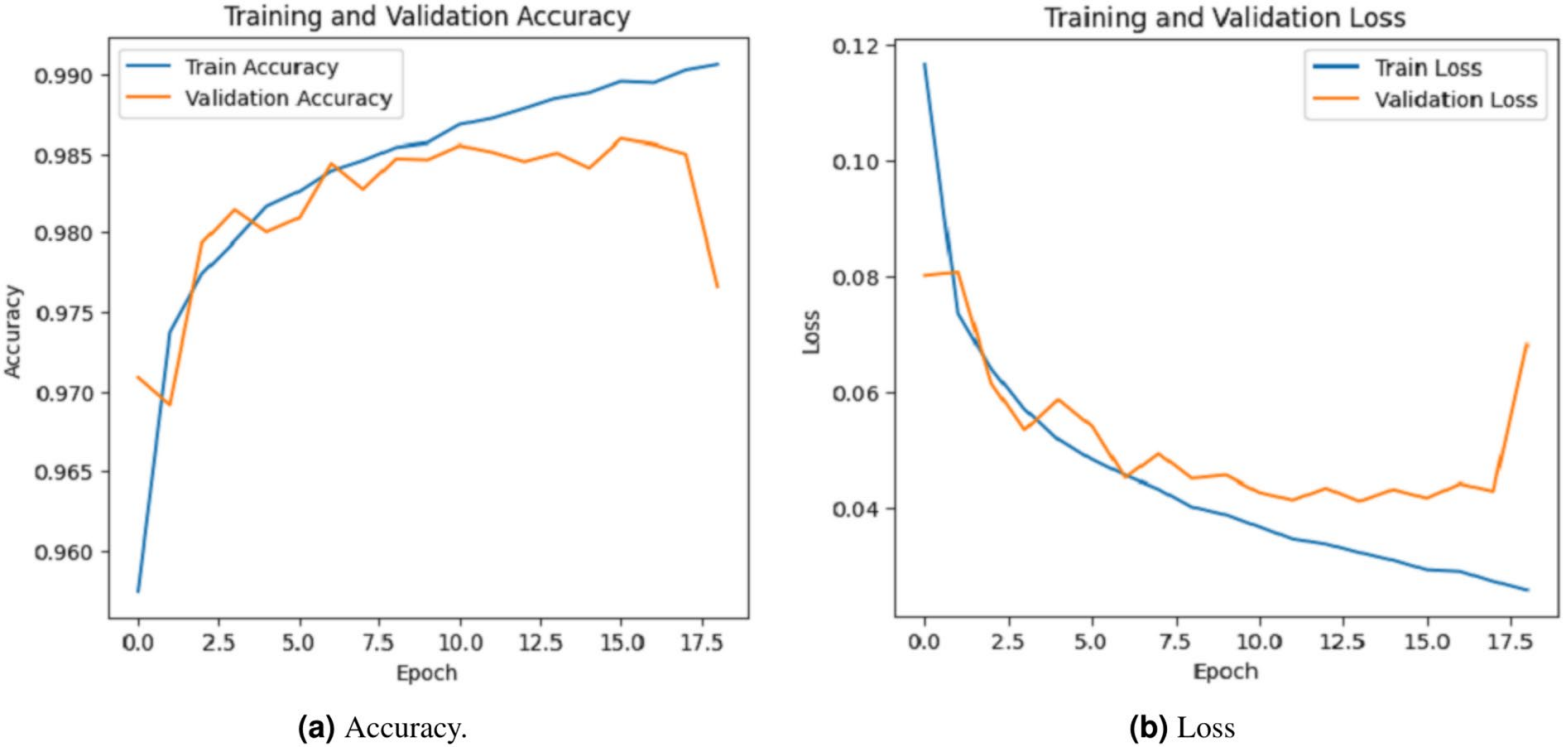
InceptionResNetV2 accuracy and loss graphs on testing MRL dataset.

**Fig. 21:**
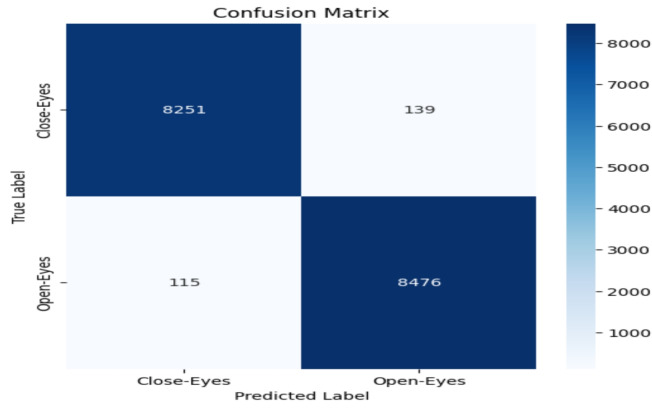
InceptionResNetV2 confusion matrix for MRL dataset.

In revolutionizing models for analyzing images, the InceptionResNetV2 model managed to score equally well to the ResNet50V2 and DenseNet169 by showcasing 98.5% for all evaluation metrics. The accuracy curve as illustrated in Fig. 20a started at a significantly low point, however with the help of training the curve managed to steadily progress upwards. The loss curve in Fig. 20b shows smooth convergence. Moreover, the confusion matrix in Fig. 21 reflects results that are similar to ResNet50V2 and DenseNet169 models which means it is also suitable for image classification.

.

#### InceptionV3

**Fig. 22:**
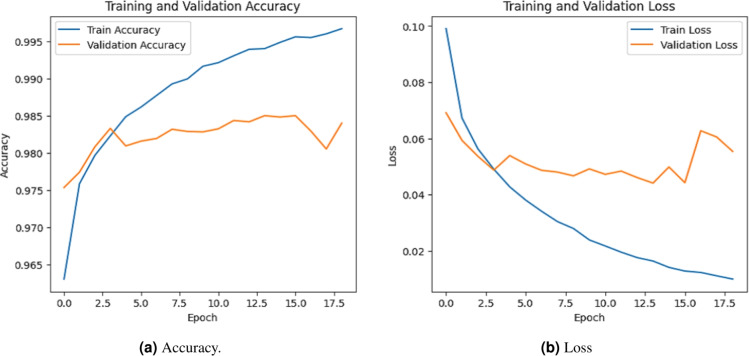
InceptionV3 accuracy and loss graphs on testing MRL dataset.

**Fig. 23:**
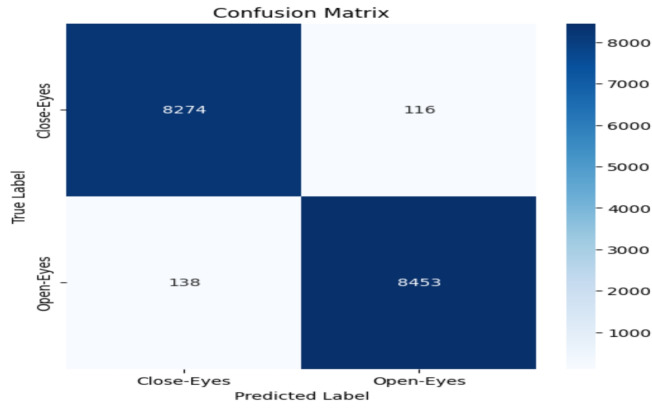
InceptionV3 confusion matrix for MRL dataset.

With accuracy, precision, recall and F1 score all being 98.5%, the InceptionV3 model achieved an accuracy of 98.5%. Figure 22a indicates a constant training period according to the accuracy graph, whilst the loss graph in Fig. 22b reinforces that there was no excessive overfitting. As seen in the confusion matrix displayed in Fig. 23, the model achieved results nearly identical to InceptionResNetV2 model, despite having a comparatively greater misclassification rate than the best of the best.

.

#### MobileNet

**Fig. 24:**
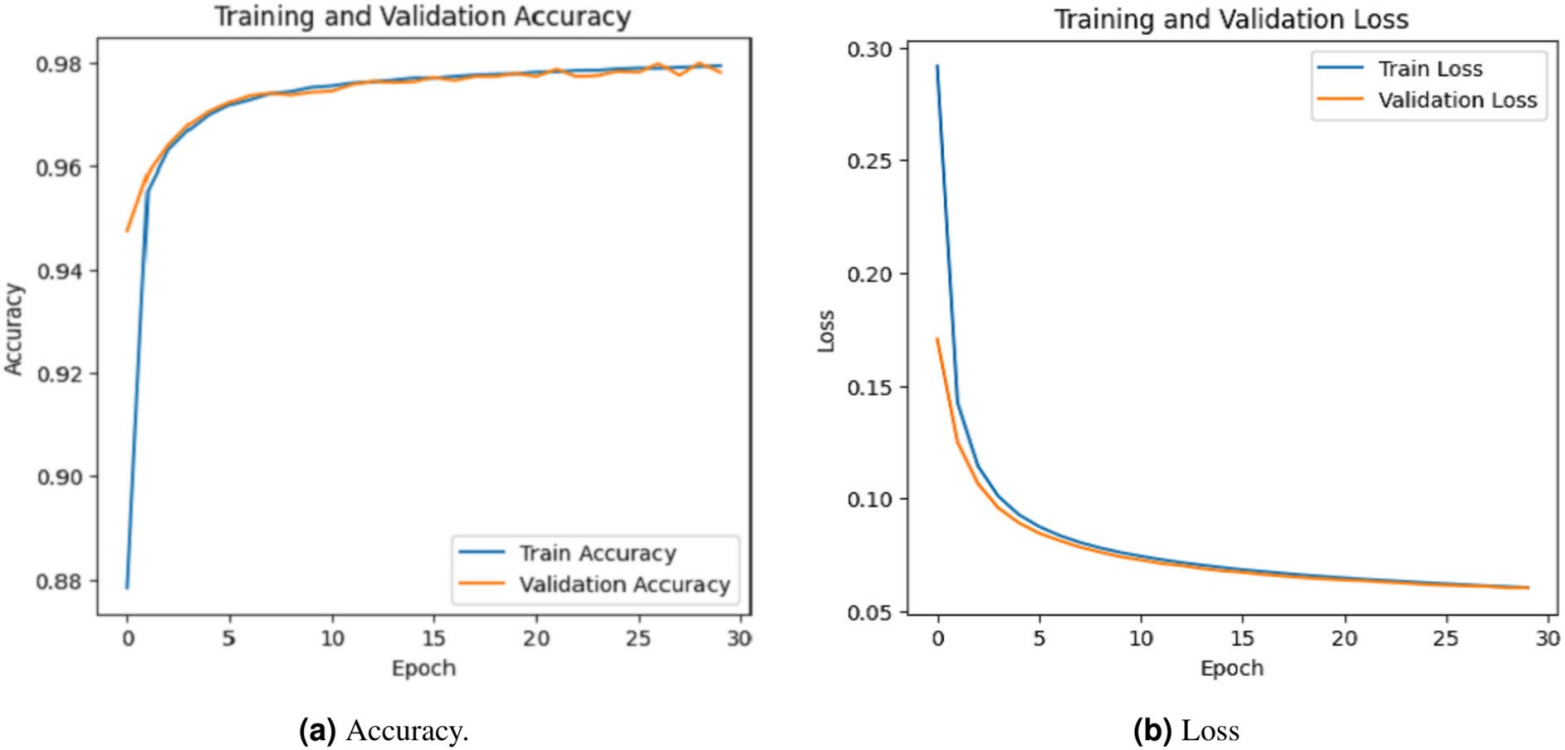
MobileNet accuracy and loss graphs on testing MRL dataset.

**Fig. 25:**
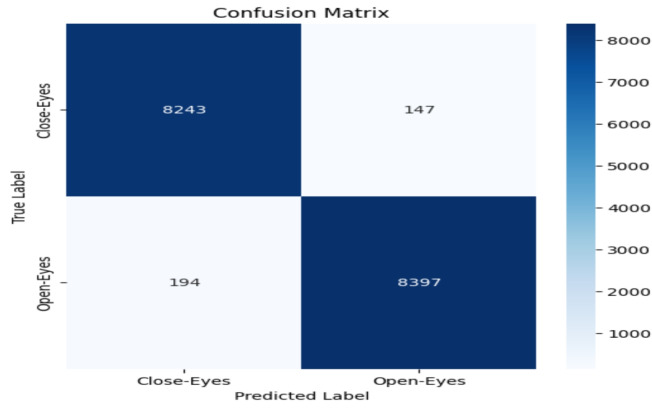
MobileNet confusion matrix for MRL dataset.

The MobileNet model was the least performing model out of the set of defined models, with 97.99% of pattern accuracy. However, it had a precision and recall of 98% with the F1 score also being equal to 98%. Figure 24a illustrates that the accuracy graph remains the same for each epoch which indicates consistency in performance, but the loss graph in Fig. 24b suggests that the convergence was effective but slower relative to the rest of the models. Emphasizing a higher error rate than the other models, the confusion matrix found in Fig. 25 suggests that mobile net was the least effective model within this context.

.

### Model learning visualization

The visualization of model learning is achieved through the use of a class activation map (CAM). CAM works by localising the different features in an image that assist in the final classification of a certain output and therefore is a significant tool for ensuring that a deep learning model is interpretable^54^. CAM makes images of lower quality that limit the model in certain features during predictions, this explains how to model predicts the states of open or close eyes and works well for drowsiness detection since it prevents the model from focusing on the surrounding areas of the eye and instead enabling them to focus on the eye itself.

**Fig. 26:**
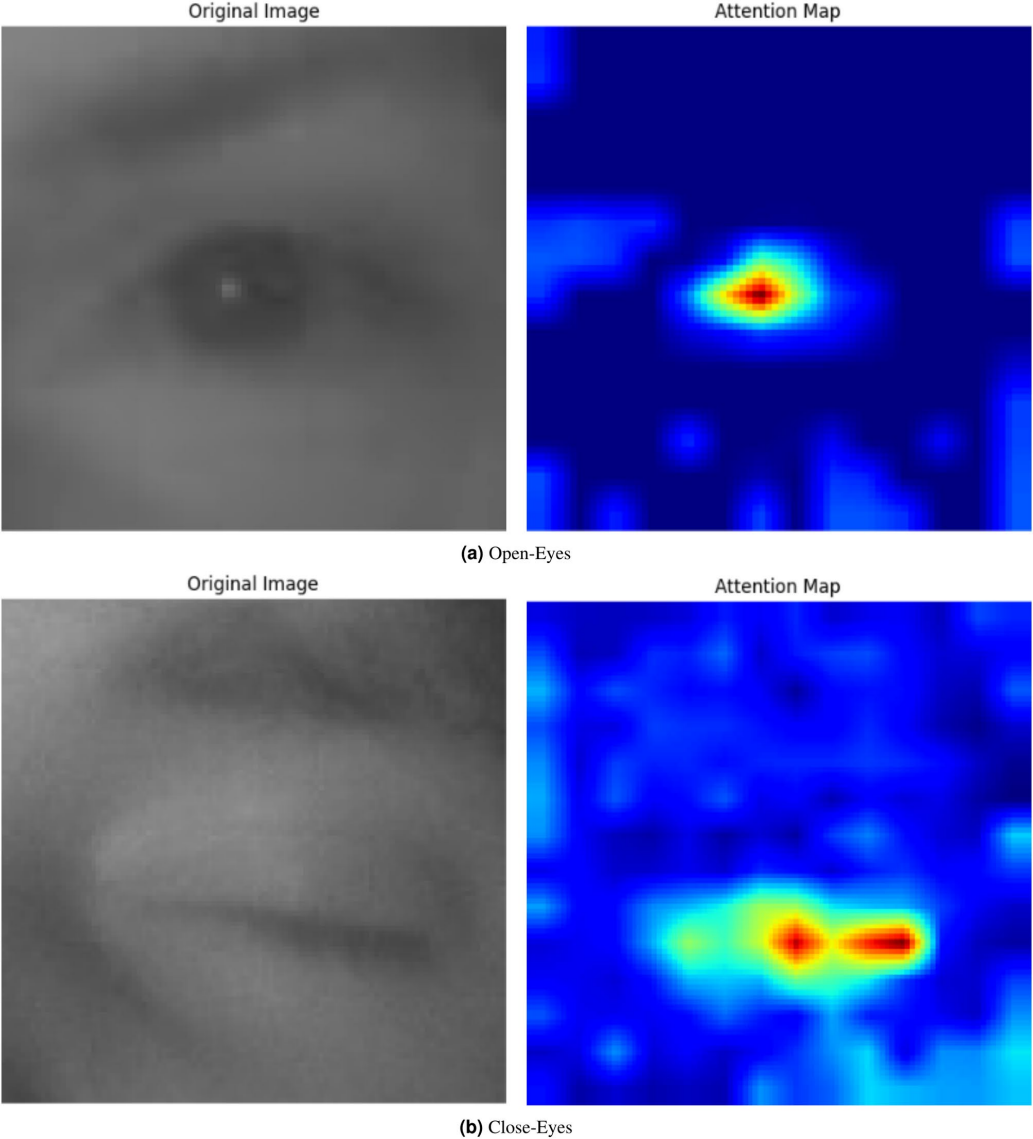
Activation map for open-eyes and close-eyes for MRL dataset.

In this research, CAM is utilized for classifying the Open-Eyes and Close-Eyes and visualizing their attention maps. The two examples in Fig. 26 demonstrate these results, in which (a) shows the original Open-Eye image together with its attention map, depicting eye-area activations, thus proving that the model can appropriately predict open eyes. Likewise, (b) shows the original Close-Eye image and its attention map with activations mostly found on the area of the closed eyelids showing that the model can accurately ascertain the state of the eye as closed With these visualizations, the model ensures reliability in practical settings such as real time driver drowsiness detection, as stated above, while still improving model interpretable by showing that the model in fact focuses on the most salient features necessary for the classification.

### Real-time deployment of drowsiness detection

**Fig. 27:**
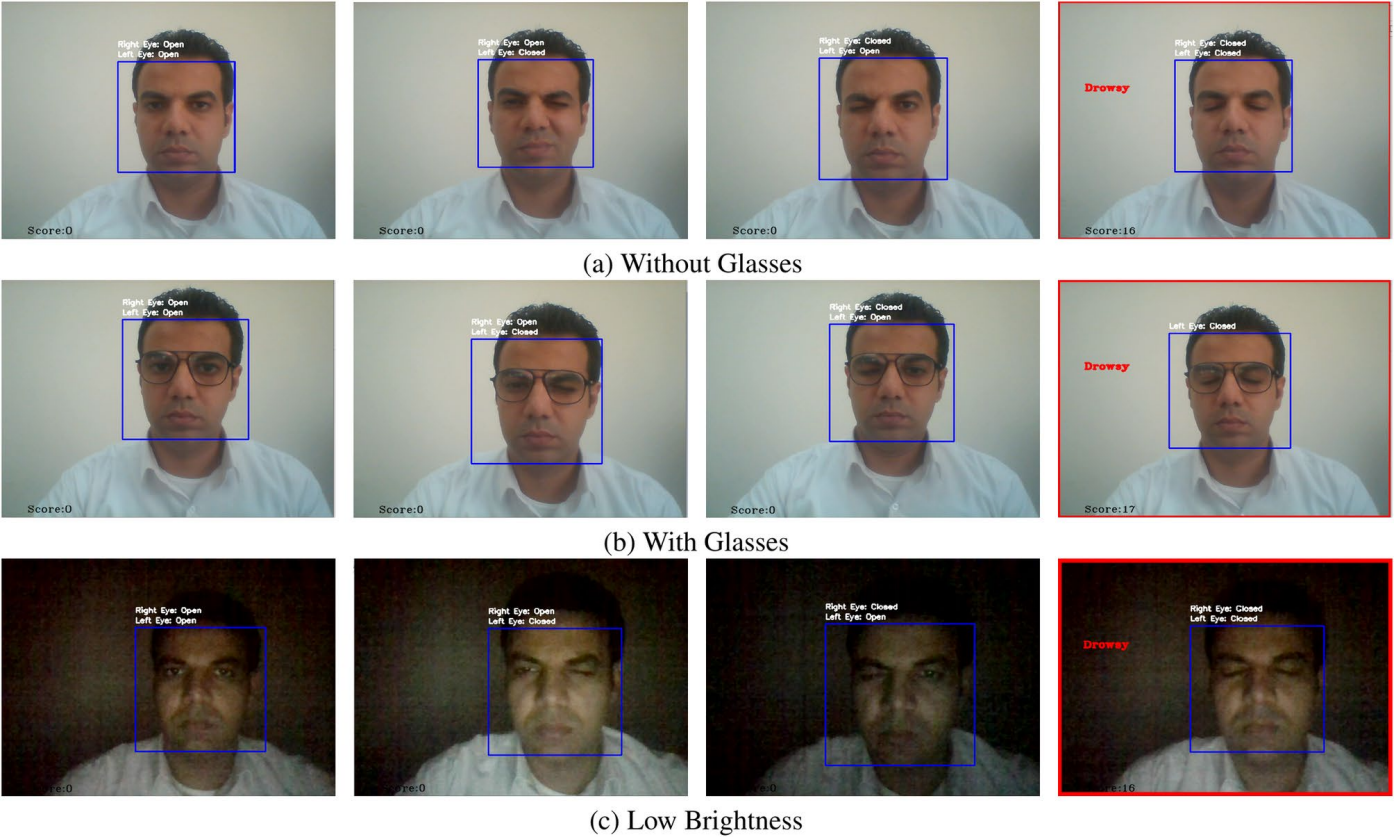
Real-time detection.

The OpenCV Library was used for detecting facial landmarks for each video frame sent by a standard webcam during the actual model deployment. To detect the right and left eye, the system first detects the face and then detects the eyes using the Haar cascade classifier. Once the model was trained, the eye states were continuously checked to see which counted frames had eyes that were closed. For this scenario, a drowsiness score was computed, and an alarm was set off when this score was more than 15. The system has been used under varying conditions, such as normal lighting without glasses, with glasses, and in low-light conditions. Fig. 27 shows the results of detection, where (a) indicates the first group of images was captured without glasses, (b) shows the second group of images was captured during average illumination with glasses, and (c) displays the last group of images captured with low brightness. All images include multiple states: open eyes, closed right eye, closed left eye, and fully closed eyes, with an alert activation when drowsiness is detected. The proposed system functions in real-time and does not require any advanced specialized hardware other than a standard webcam, which makes it easier to implement on desktop computers, mobile devices, and other platforms.

In addition to accuracy metrics, we performed a comprehensive evaluation of model complexity to assess the efficiency and practical applicability of the proposed framework. As shown in Table 11, we compared the number of trainable parameters and average inference time per image across all models. Transformer-based models such as ViT and Swin Transformer achieved the highest accuracy, significantly outperforming all other models in classification performance. However, these models are relatively heavier and require further optimization to improve inference time for real-time applications. On the other hand, transfer learning models such as MobileNet and VGG19 demonstrated excellent real-time performance due to their lightweight architecture and faster inference time but showed relatively lower accuracy compared to transformer models. This trade-off highlights a key insight: while transformers are optimal for accuracy-critical scenarios, transfer learning models remain a practical solution where real-time constraints are stringent. Furthermore, through optimization techniques such as pruning or quantization, transformer models can be adapted for deployment on edge devices. These findings underscore the need for balancing accuracy and efficiency to ensure scalable and robust real-time driver drowsiness detection.

**Table 11:** Model performance and complexity comparison.

Model	Accuracy (%)	Parameters	Average inference time (ms/frame)	Average FPS
ViT transformer	99.15	85,800,194	**1021.83**	0.98
Swin transformer	99.03	27,914,108	**472.8**	2.12
InceptionV3	98.5	23,867,553	**136.33**	7.35
InceptionResNetV2	98.5	55,851,105	**122.8**	8.14
MobileNet	98	3,208,001	**55**	18.18

## Comparative study

Eye state recognition has faced obstacles in previous studies. More seriously some constraints in resources appear to be difficult not only to improve but also to optimize accuracy with timing. Regarding the work presented in^25^ which employs a dual CNN ensemble else, there exist a few other approaches which highlight a significant increase in speed over previous architecture. Their system has achieved unprecedented accuracy of 97.99% on the ZJU dataset which surpassed the previous achievement by 0.79% at 97.20%. Their method based on transfer learning and fine tuning circumvents the issue of overfitting when dealing with small data sets and unlike other models there is no emphasis in parameters or lower recognition rate. Unlike previous solutions such as those raising the problem of gaining speed at the sacrifice of accuracy, or others optimizing for computational efficiency, this system has been able to work efficiently with embedded systems while maintaining high accuracy for multiple datasets concurrently.

In^28^, authors proposed the use of deep mastering adaptation mapping of convolutional neural networks’ practices. To this vision, have been included three CNN models, namely VGG16, VGG19, and new models of 4D. MRL Eye dataset was used to develop teaching sets for the 4D model, which was previously designed only for the assessment of driver drowsiness in more advanced quite dark conditions. The 4D model also had a better predictive performance than both the VGG16 and VGG19 models. This information describes a practical integrated system of a driving simulator comprising an intelligent system that estimates the driver visual state in order to detect drowsiness and provide an appropriate warning for road safety.

In this article^31^, the authors conducted an in-depth study with practical applications from their findings of a CNN based real-time, non-invasive drowsiness detection system that analyzes a driver by utilizing video footage obtained from a camera that is installed within the vehicle to assess the driver’s fatigue. In the system, yawning, eye states and various facial expressions are classified in order to detect signs of fatigue. The model is based off of a dependable architecture that has been trained with a wide range of datasets containing images taken under different lighting conditions and angles. It also uses Haar cascade classifiers for facial detection alongside fatigue detection technology. Furthermore, the study observes a 96.54% testing accuracy, confirming that CNN models improve safety on roads by decreasing the amount of collisions caused by drowsy drivers’ inactivity.

Trying to improve over other drowsiness detection methods, the authors in^18^ embarked on a quest that involved drowsiness detection deployment through a mobile application which makes use of an amalgamation of transfer learning and CNN. As for their theoretical research, the effectiveness of their mobile app was thoroughly evaluated to establish its practicality which involved gathering numerous datasets. The mobile application operates under the hypothesis that the drowsiness detection system is expected to be between 96 and 98% with the MRL dataset.

The transformer-based model proposed in this paper surpasses all existing methods in the Open-Eyes Vs Close-Eyes classification problem. As opposed to the CNN-based algorithms, the Use of the transformer structures improves the process of feature extraction by paying attention to only relevant features and not unnecessary ones. Testing results with dataset MRL validate our proposed model as noted from the remarkable 99.15% testing accuracy attained using a ViT transformer or 99.03% when using Swin Transformer and all 99 percent of precision, recall and F1 score in respect to most other models tested. The proposed model outperformed all other seven models tested with all the fine-tuned hyperparameters in accuracy as well as robustness. While a number of these models including VGG19, ResNet50V2, MobileNetV2, and Inception V3, DenseNet169, InceptionResNetV2 yielded high scores for accuracy none were able to exceed the efficiency provided by a Transformer based model. This increase in performance reiterates through evidence the strengths associated with self attention mechanisms targeting the distinct eye states feature proving the superiority of the Transformer architecture especially in real time scenarios involving drowsiness detection. The comparison of the results with the previous related works results is displayed in Table 12.

**Table 12:** Comparison of results of previous literature results on MRL dataset.

Ref	Year		Model	Accuracy (%)	Precision (%)	Recall (%)	F1-score (%)
^25^	2022		Dual CNN Ensemble (DCNNE)	98.98	–	–	–
^28^	2023		VGG16	95.93	93.15	93.87	–
			VGG19	95.03	94.82	95.47	–
			4D	97.53	97.35	97.06	–
^31^	2024		VGG16	95.85	–	–	- -
			CNN	96.54	–	–	- -
^18^	2024		CNN	96	around 94:98	around 94:98	around 94:98
			MobileNetV2	97	99.42	92.32	95.61
			InceptionV3	98	Around 97:98	Around 97:98	Around 97:98
			Proposed swin transformer	99.03	99	99	99
			Proposed ViT transformer	99.15	99	99	99

## Discussion

The current study demonstrates the strong potential of transformer-based models, particularly ViT and Swin Transformer, in detecting driver drowsiness with high accuracy and generalizability. The ViT model achieved 99.15% accuracy on the MRL dataset and retained robust performance on unconstrained datasets such as CEW and NTHU-DDD. These results show a marked improvement over traditional CNN-based models (e.g., ResNet50V2 and InceptionV3), particularly in handling occlusions, lighting variability, and facial expressions. The transformer models’ self-attention mechanisms were instrumental in capturing nuanced spatial features like partial eye closure and facial angle variability. While transformer architectures are computationally more demanding, their superior performance in safety-critical applications such as real-time drowsiness detection justifies the computational trade-off. The results suggest that the proposed system can be effectively implemented in real-time driver assistance platforms.

One notable limitation of this study is the reliance on the MRL Eye Dataset as the primary source for training the deep learning models. While the MRL dataset offers a balanced and well-structured set of close-up eye images, its constrained environment - including grayscale images, close-up framing, and limited variability in lighting and facial characteristics - may restrict the generalizability of the trained models to real-world conditions. Despite these limitations, MRL was chosen due to its large volume of well-labeled eye state images, which provide a reliable foundation for training and benchmarking classification models. To mitigate the risk of overfitting to this controlled dataset and to assess the robustness of our models, we extended our evaluation to include two additional datasets: NTHU-DDD, which provides annotated video sequences under diverse lighting, pose, and behavioral conditions; and CEW, which includes unconstrained facial images with variations in head orientation, facial expressions, and occlusions such as glasses. These supplementary evaluations helped test the adaptability of the proposed models beyond the conditions present in MRL and confirmed their competitive performance in more diverse, real-world scenarios. Nonetheless, future work will aim to further enhance generalizability through training on larger, real-world datasets and integrating techniques such as domain adaptation and field-deployable validation. We also plan to combine these datasets in real-time applications to further improve robustness and practical deployment.

While the ViT model achieved a high classification accuracy of 99.15% on the MRL dataset, and similarly high results were obtained on NTHU-DDD and CEW, these metrics do not guarantee equivalent performance in real-world driving environments. Real-world conditions introduce unpredictable variables such as poor or rapidly changing lighting, head movements, facial occlusions due to sunglasses or hair, and differences in driver facial features and skin tones. Although the CEW and NTHU-DDD datasets were included specifically to simulate some of these conditions - such as varied facial angles, occlusions, and lighting - it remains a limitation that the system has not yet been field-tested in uncontrolled environments. Future work will involve deploying the system in real vehicles under various real-world conditions to assess its robustness and make necessary model adjustments. Additionally, incorporating adaptive learning or domain adaptation techniques may further help mitigate performance drops caused by environmental variability.

Another limitation of the current study lies in the granularity of the labels within the MRL dataset. Since it only includes static images categorized into binary eye states - Open and Closed - the models trained on this dataset are inherently constrained in their ability to detect more subtle or gradual signs of drowsiness, such as heavy blinking, partial eye closure, or slow eyelid drooping. These nuances often precede full eye closure and are critical for early intervention. To partially address this issue, we incorporated the NTHU-DDD dataset in our evaluation, which includes annotated behavioral cues like slow blinking, yawning, and nodding across video frames. However, fully capturing these transitional or temporal features requires future work involving sequential modeling techniques (e.g., RNNs or LSTM-based approaches) and datasets with continuous drowsiness annotations. Expanding to such models would enable the system to respond more sensitively to early drowsiness indicators, improving real-world applicability and safety impact.

While transfer learning and pre-trained models such as ViT, Swin Transformer, and VGG19 enabled significant performance improvements in this study, these models come with inherent limitations. Since they are initially trained on large-scale general datasets (e.g., ImageNet), their learned features may not fully capture the specific nuances of driver drowsiness behavior, particularly under unique environmental or cultural contexts. Moreover, highly complex pre-trained models can sometimes overfit to training data when fine-tuned on smaller or less diverse datasets, potentially reducing adaptability in real-world deployment. To mitigate this, we employed data augmentation and cross-dataset validation using CEW and NTHU-DDD. However, future research could explore domain-specific model pretraining or self-supervised approaches tailored to driver monitoring systems, improving robustness and generalization across unseen environments.

Compared to prior models that rely primarily on CNN architectures, such as VGG19 or Inception-based networks, the proposed transformer-based models - particularly ViT and Swin Transformer - demonstrated significantly higher robustness across datasets with real-world variability (e.g., CEW and NTHU-DDD). The ViT model achieved 99.15% accuracy on the MRL dataset and retained strong performance on more unconstrained data from CEW (97.25%), where models like ResNet50V2 and InceptionV3 showed lower generalization. This suggests that the self-attention mechanism in transformers allows for better spatial feature representation, especially when dealing with subtle visual cues like partial eye closure or facial occlusion. While transformer models are generally more computationally intensive than traditional CNNs, their improved accuracy in complex conditions justifies the trade-off for real-time safety-critical applications. Moreover, unlike models tailored to a single dataset or environment, our approach generalized well across multiple benchmark datasets, highlighting its adaptability and robustness in diverse scenarios. Future comparative studies will aim to evaluate these models in field conditions and compare not only accuracy but also latency and resource efficiency for embedded deployment.

To address concerns about generalizability and real-world applicability, we expanded our evaluation to include two challenging and diverse datasets: NTHU-DDD and CEW. These datasets present complex driving-related conditions such as varied head poses, facial expressions, occlusions (e.g., sunglasses), and lighting conditions, making them suitable for assessing the robustness of our proposed framework. The high performance of our model on these datasets supports its adaptability to more realistic settings. Nonetheless, we recognize that deeper behavioral analysis - such as incorporating multimodal inputs like head pose estimation, facial emotion recognition, or yawning detection - would strengthen the system’s ability to model driver fatigue more comprehensively. In future work, we plan to expand the model to integrate these cues, enabling a richer representation of driver state and supporting more accurate, context-aware interventions for road safety.

While transformer-based models demonstrated superior accuracy, they were more computationally intensive compared to transfer learning models. In contrast, transfer learning models offered faster inference but slightly lower accuracy. This trade-off highlights the importance of selecting the appropriate model based on the deployment environment-prioritizing accuracy for high-performance systems and speed for real-time, resource-constrained applications.

## Conclusion and future directions

This work presents a highly effective deep learning-based framework for real-time driver drowsiness detection, achieving superior results through the application of state-of-the-art transformer architectures and transfer learning models. The proposed system, which classifies eye states into “Open-Eyes” and “Close-Eyes” using the MRL Eye Dataset, demonstrated exceptional performance, with the Vision Transformer (ViT) and Swin Transformer models achieving remarkable accuracies of 99.15% and 99.03%, respectively. These results significantly outperform existing methods, highlighting the superiority of transformer-based models in capturing complex spatial dependencies and extracting relevant features for drowsiness detection. The integration of Haar Cascade classifiers for real-time face and eye detection, coupled with a drowsiness scoring mechanism, ensures timely and accurate alerts, enhancing road safety by mitigating the risks associated with fatigue-related accidents. The proposed framework was rigorously tested under various lighting conditions, including normal, low-light, and scenarios involving glasses, demonstrating its robustness and adaptability for real-world deployment. The use of Class Activation Mapping (CAM) further enhanced model interpretability, ensuring that the system focuses on critical eye regions for accurate classification. Compared to existing methods, the proposed framework offers a significant improvement in accuracy, precision, recall, and F1-score, making it a reliable and efficient solution for real-time drowsiness detection. Future work will focus on optimizing the system for deployment on resource-constrained embedded devices, exploring multi-modal approaches that integrate additional physiological signals, and extending the dataset to include more diverse driving scenarios. By addressing these challenges, the proposed framework has the potential to significantly contribute to the development of advanced driver assistance systems, ultimately reducing the incidence of drowsiness-related accidents and improving overall road safety. While our real-time tests included glasses and low-light scenarios through 3 different datasets, broader dataset diversity is needed for universal deployment. Future work will integrate multi-modal inputs (e.g., infrared cameras for low-light) and federated learning to adapt to unseen environments. The superior results achieved in this work underscore the effectiveness of the proposed approach and its potential for real-world application. Current drowsiness detection systems, including ours, primarily react to observable symptoms (e.g., eye closure). However, proactive prevention requires identifying pre-fatigue cues (e.g., increased blink duration, gaze instability) before severe drowsiness occurs. While our transformer models achieve 99.15% accuracy in classifying eye states, future iterations must integrate temporal dynamics and multi-modal signals (e.g., steering patterns, head nods) to predict drowsiness onset.

## Data Availability

The paper has a datasets available in the Kaggle repository MRL Dataset: https://www.kaggle.com/datasets/imadeddinedjerarda/mrl-eye-dataset NTHU-DDD Dataset: https://www.kaggle.com/datasets/banudeep/nthuddd2 CEW Dataset: https://www.kaggle.com/datasets/ahamedfarouk/cew-dataset
